# Xenogeneic equine stem cells activate anti-tumor adaptive immunity in a 4T1-based intraductal mouse model for triple-negative breast cancer: proof-of-principle

**DOI:** 10.3389/fimmu.2023.1252374

**Published:** 2023-10-20

**Authors:** Jonas Steenbrugge, Glenn Pauwelyn, Kristel Demeyere, Nausikaa Devriendt, Hilde de Rooster, Niek N. Sanders, Jan H. Spaas, Evelyne Meyer

**Affiliations:** ^1^ Laboratory of Biochemistry, Department of Veterinary and Biosciences, Faculty of Veterinary Medicine, Ghent University, Merelbeke, Belgium; ^2^ Cancer Research Institute Ghent (CRIG), Ghent, Belgium; ^3^ Boehringer Ingelheim Veterinary Medicine Belgium, Evergem, Belgium; ^4^ Small Animal Department, Faculty of Veterinary Medicine, Ghent University, Merelbeke, Belgium; ^5^ Laboratory of Gene Therapy, Department of Veterinary and Biosciences, Faculty of Veterinary Medicine, Ghent University, Merelbeke, Belgium; ^6^ Department of Morphology, Imaging, Orthopedics, Rehabilitation and Nutrition, Faculty of Veterinary Medicine, Ghent University, Merelbeke, Belgium; ^7^ Boehringer-Ingelheim Animal Health USA, Athens, GA, United States

**Keywords:** triple-negative breast cancer, intraductal model, immunotherapy, xenogeneic, equine stem cells

## Abstract

Triple-negative breast cancer (TNBC) remains difficult to treat, especially due to ineffective immune responses. Current treatments mainly aim at a cytotoxic effect, whereas (stem) cell therapies are being investigated for their immune stimulatory capacities to initiate the anti-tumor immunity. Here, a thoroughly characterized, homogenous and non-tumorigenic mixture of equine mesenchymal stem cells (eMSCs) harvested from horse peripheral blood as innovative xenogeneic immunomodulators were tested in a 4T1-based intraductal mouse model for TNBC. The eMSCs significantly reduced 4T1 progression upon systemic injection, with induction of inflammatory mediators and T-cell influx in primary tumors, already after a single dose. These xenogeneic anti-cancer effects were not restricted to MSCs as systemic treatment with alternative equine epithelial stem cells (eEpSCs) mimicked the reported disease reduction. Mechanistically, effective eMSC treatment did not rely on the spleen as systemic entrapment site, whereas CD4^+^ and CD8α^+^ T-cell infiltration and activation were critical. These results show that eMSCs and potentially also other equine stem cell types can be a valuable TNBC treatment strategy for further (pre)clinical evaluation.

## Introduction

Triple-negative breast cancer (TNBC) is one of the most aggressive breast cancer subtypes with poor prognosis and few therapeutic options besides chemotherapy (1). Recently, innovative immunotherapies such as immune checkpoint blockers (ICBs) have provided further improvement in patient outcome. These ICBs activate the tumor cell killing capacity of mainly CD8^+^ T-cells and natural killer (NK) cells in TNBC patients, inducing durable anti-tumor effects (2). Yet, 80-90% of TNBC patients remain unresponsive to ICBs related to their immunosuppressive tumor microenvironment (TME) and low tumor immunogenicity (3–5).

An intriguing and novel alternative of boosting the anti-tumor immunity in TNBC patients is the use of xenogeneic (stem) cell types. More specifically, Huang et al. recently reviewed the novel applications of xenogeneic pig-derived cells (6) and described one study on porcine mammary-specific glandular cells to treat a 4T1-based mouse model for TNBC (7). Apart from the need for fresh porcine mammary gland tissue for cellular isolation, the xenogeneic cell types were injected intratumorally, restricting their anti-tumor activity to the primary tumor site and potentially leaving metastatic lesions untreated. Yet, literature on the anti-cancer application of xenogeneic cells is still scarce and their optimal source and injection route as well as their immunological mode-of-action and potential side effects are not fully known. Another study by Wei et al. described the use of intraperitoneal (i.p.) injected xenogeneic endothelial cell lines that provided antibody-mediated blood vessel reduction in primary tumors (8), albeit without durable stimulation of anti-tumor immunity. Moreover, the hypoxic environment that accompanies blood vessel reduction in the primary tumor may induce compensatory proliferative pathways that fuel further malignancy (9–11).

In the current preclinical study, we report the use of equine mesenchymal stem cells (eMSCs) as previously unexplored xenogeneic cell type with advantages towards isolation and cultivation as well as evoking anti-tumor responses in the context of TNBC. Indeed, besides their ease of collection in large numbers through venopuncture from carefully selected and pathogen-free donor horses, eMSCs can also be rapidly expanded *in vitro* due to their well-characterized multipotency (12–14). Using a well characterized 4T1-based intraductal mouse model for human TNBC (15), our results show that systemic injection of a low and even single eMSC dose provides durable stimulation of anti-4T1 tumor adaptive immunity, resulting in significant disease reduction and metastatic eradication. The observed disease-reducing effects were also not restricted to eMSCs but could be mimicked by other equine stem cell types such as equine epithelial stem cells (eEpSCs). These novel results on equine stem cells in general should now be further explored in other (pre)clinical models.

## Materials and methods

### 4T1 cell culture

The 4T1-luc mammary tumor cell line resembling human TNBC metastasis and constitutively expressing firefly luciferase was a kind gift from Prof. Clare Isacke (Breakthrough Breast Cancer Research Centre, London, UK). 4T1-luc cells were cultured in Dulbecco’s Modified Eagle’s Medium (DMEM) supplemented with 10% heat-inactivated fetal bovine serum (FBS), 100 U/ml penicillin and 100 μg/ml streptomycin (Thermo Fisher Scientific, Waltham, MA, USA) in culture flasks at 37°C and 5% CO_2_. Cell cultures were negative for mycoplasma and bacterial contamination based on PlasmoTest^TM^ results (Invivogen, San Diego, USA).

### Equine stem cell isolation, culturing and characterization

The eMSCs and eEpSCs, respectively, were isolated from venous blood collected from the vena jugularis and epidermal skin sample of a donor horse. Animal manipulations necessary for this isolation were approved by an independent ethics committee approved by the Flemish government (recognition number: LA1700607; EC 2018-002 and 2014-001) and Faculty of Veterinary Medicine, Ghent University (EC 2014-020). As described previously, the donor horses were first tested for multiple transmittable diseases (16). A subsequent enzymatic dissociation step on the skin sample was necessary to obtain an eEpSC-containing suspension. The cells were then cultivated in a Good Manufacturing Practice (GMP)-certified production site (BE/GMP/2018/123) according to GMP-guidelines until passage (P) 5. In order to obtain a highly pure eEpSC population, several purification rounds were performed with final plating of the cells on ultralow-attachment plates. The eMSCs and eEpSCs were further characterized on viability, morphology, presence of cell surface markers, population doubling time (acceptance criteria are between 0.7 and 3.0) and, only in the case of eMSCs, trilinear differentiation. Evaluation of the presence (CD29, CD44, CD90 for eMSCs; CD49f, p63 for eEpSCs) and absence (Major Histocompatibility Complex (MHC) II, CD45 and marker for monocytes and macrophages for eMSCs; CD105, MHCI and II for eEpSCs) of specific cell surface markers was accomplished by flow cytometry as previously described (14). Consequently, the cells were cultivated until P10, trypsinized, resuspended in DMEM low glucose with 10% dimethylsulfoxide (DMSO) and stored at -80°C in cryovials until further use.

### Intraductal 4T1 cell injections and treatment

Mouse experiments were performed according to Good Scientific Practice-principles and approved by the Ethical Committee (EC) of the Faculty of Veterinary Medicine, Ghent University (EC 2020-030 and 2021-018).

Eight-week (w)-old female and male BALB/c mice were mated and pups were weaned 12-14 days (d) post-parturition. One hour (h) after weaning, lactating females were intraductally inoculated in the third mammary gland pair with 5 × 10^4^ 4T1-luc cells suspended in a 100 μl mixture of PBS and Matrigel^®^ (1:10; Corning, Bedford, MA, USA) under inhalation anesthesia and analgesia as described previously (15). For intravenous (i.v.) treatment, eMSCs or eEpSCs were dissolved in DMEM at 3x10^5^ per 100 μl and injected through the tail vein using a 29G insulin needle. I.v. injections with DMEM only were used as a negative (sham) control. Antibody treatments for depletion of CD4^+^ (clone GK1.5) and CD8α^+^ T-cells (clone YTS169.4) or rat IgG2b isotype controls (clone LTF-2) were purchased from BioXCell (West Lebanon, NH, USA) and diluted (200 μg/100 μl) in InVivoPure dilution buffer (BioXCell) at pH6.5 (for anti-CD4) or pH7 (for anti-CD8α and isotype control) for i.p. administration.

### Analysis of disease progression

4T1 primary tumor growth was determined through weekly measurement of primary tumor volumes and *in vivo* imaging. Primary tumor volume measurements relied on the use of a digital caliper to determine length, width and height of the primary tumor for subsequent volume calculation (length x width x height). The *in vivo* imaging technique to determine primary tumor growth relied on the detection of the 4T1-luc-derived bioluminescence in primary tumors using the IVIS lumina III system (PerkinElmer, Zaventem, Belgium). For the *in vivo* imaging procedure, mice were i.p. injected with D-luciferin (2 mg/100 μl PBS; Gold Biotechnology, St. Louis, MO) and placed in the IVIS system 10 min later under inhalation anesthesia as described previously (17). For *ex vivo* imaging, primary tumors, spleens and metastases-bearing organs were quickly isolated from the mice following terminal sedation and sacrification through cervical dislocation, and placed in the IVIS system to capture bioluminescent signals (17). IVIS measurements and subsequent analysis were performed using the living image software 4.7.2.

### ^99m^Tc labeling and SPECT/CT tracing of eMSCs

The ^99m^Tc labelling of the eMSCs was performed as recently described (18). Briefly, 9 x 10^5^ eMSCs were pelleted and resuspended in saline mixed with SnCl2 and freshly eluted ^99m^TcO4 (Sigma Aldrich, US). The preparation was incubated for 30 minutes (min) at room temperature (RT), after which the eMSCs were washed with DMEM and subsequently suspended to a concentration of 3 x 10^5^ eMSCs/100 µl DMEM.

All mice were food deprived for minimum 12 h before injection of the ^99m^Tc-eMSCs. For preparing the injection line and during injection of the tracer (23.6 ± 5.5 MBq) via the tail vein, mice were put under inhalation anesthesia using isoflurane (5% induction, 2% maintenance). SPECT scans were acquired for each mouse on a U-SPECT-II system (MILabs, The Netherlands) and were immediately followed by a CT acquisition on the same device. For each animal, SPECT/CT scans were performed at 4 different time points following i.v. tracer injection: 1 h, 4 h, 7 h and 22 h. The SPECT acquisitions were acquired using a multi-pinhole collimator containing 75 pinholes with 1 mm pinhole diameter. Five bed positions were acquired to obtain total body mouse SPECT images. The total acquisition time was 45 min (9 bed positions of 5 min/bed position). The following CT acquisition parameters were used to obtain total body mouse CT images: 50 kV tube voltage, 600 µA tube current, 200 projections over 360 degrees, 1 bed position, averaging 1, total acquisition time 3 min and 24 seconds (sec).

The acquired SPECT data were iteratively reconstructed using the Ordered Subsets Expectation Maximization (OSEM) algorithm, delivered by the manufacturer of the U-SPECT-II system. A total of 4 iterations was used in combination with 16 subsets. The reconstructed voxel size was 0.75 mm, the photopeak window for ^99m^Tc was set to 140 keV ± 20%. The acquired CT projection images were reconstructed by an analytic back projection algorithm, delivered by the manufacturer of the U-SPECT-II system. The reconstructed SPECT and CT images were automatically co-registered, stored as NIfTI format and imported into AMIDE (A Medical Image Data Examiner). Finally, the %ID/g was calculated at each time point for the primary tumor, lungs, liver, spleen and bladder.

### Protein analysis

Lysates were prepared from isolated primary tumors, axillary lymph nodes, lungs and spleen as previously described (15). Following protein concentration determination, nine cytokines (granulocyte-colony stimulating factor (G-CSF), interferon (IFN)-γ, interleukin (IL)-1β, IL-4, IL-6, IL-10, monocyte chemoattractant protein (MCP)-1, macrophage inflammatory protein (MIP)-2 and tumor necrosis factor (TNF)-α) were quantified in all lysates (25-50 μg protein) using the Luminex Multiplex Assay (ProcartaPlex from Thermo Fisher Scientific) according to the manufacturer’s instructions. Enzyme-linked immunosorbent assay (ELISA) was used to measure transforming growth factor (TGF)-β1 levels (Thermo Fisher Scientific) in the lysates according to the manufacturer’s instructions.

### Immunohistochemistry

Isolated tissues were fixed in buffered 3.5% formaldehyde for 24 h at RT and embedded in paraffin wax. hematoxylin & eosin (H&E) staining was performed on 5 µm thick deparaffinized sections. For immunohistochemical staining, antigen retrieval was performed on 3-5 μm thick deparaffinized sections either with citrate buffer (pH 6, 10 mM tri-sodium citrate (Santa Cruz Biotechnology, Heidelberg, Germany) for Ki67, CD45, CD163, CD3ϵ, granzyme B), or with Tris-ethylenediaminetetraacetic acid (EDTA) buffer (pH 9, 10mM Tris, 1mM EDTA (Thermo Fisher Scientific) for CD4, CD8a and NCR-1) using a pressurized Decloaking Chamber NxGen (Biocare Medical, CA, USA). As previously described (15, 17), the slides were subsequently incubated on an orbital shaker in a closed microscope box with tris-buffered saline (TBS, Biocare Medical)-wetted tissue paper for all blocking, rinsing and staining steps. For visualization of HRP-positive staining, slides were treated with a 3,3′-diaminobenzidine (DAB)-containing buffer (Dako). Counterstaining with hematoxylin was applied, followed by dehydration and mounting of the slides. Quantification of positive staining was established using either color deconvolution followed by automatic counting in ImageJ. Ki67 proliferation indices were determined using ImageJS (19).

### Splenectomy of 4T1 tumor-bearing mice

Following anesthesia and disinfection of the skin using an iodine solution (Povidone-Iodine, Ecuphar), a small incision (5-10 mm) was made in the ventral midline caudal to the xiphoid of the abdomen of the 4T1 tumor-bearing mice. A small stab incision was made in the linea alba and lengthened by blunt opening of the scissors. The spleen was extoriated by lifting the omentum and splenic vessels were cauterized at the hilum. Analgesia (PBS-diluted buprenorphine) was provided in the abdomen prior to closure, and mice recovered under an infrared lamp after surgery. Mice undergoing sham splenectomy also received similar surgical incisions and the spleen was also exteriorized, but, after inspection, carefully placed back in the abdominal cavity.

### Flow cytometric immunophenotyping

Primary tumors and spleens were isolated from the 4T1 tumor-bearing mice and stored in MACS tissue storage solution (Miltenyi Biotec, Leiden, The Netherlands) or directly placed in DMEM used for 4T1 cell culture. Briefly, primary tumors were transferred into a tumor dissociation enzyme mix (Miltenyi Biotec) and processed on a gentleMACS Dissociator, followed by incubation for enzymatic digestion and filtering through a 70 μm strainer. Splenic fragments were aspirated and pushed through a 22G ¼ needle, after which a 70 μm strainer was applied and blood cell lysis was performed by applying ACK (Ammonium-Chloride-Potassium) lysing buffer as described previously (17). The splenic and primary tumor cell solution was subsequently stained with Viobility 488/520 Fixable dye (Miltenyi Biotec) for exclusion of dead cells. Next, a cocktail composed of anti-mouse REAfinity antibodies (all from Miltenyi Biotec) diluted in FACS buffer (containing PBS with 1% bovine serum albumin (BSA), 2.5mM EDTA and 0.01% sodium azide) was applied and analyses were performed on a flow cytometer. Data were processed using CytExpert v2.0.0.153 software (Beckman Coulter, Inc., California, USA).

### Statistical analysis

Statistics were performed using Prism (Graphpad). Data normalization was performed through log^10^ normalization when appropriate. P-values were calculated by Student’s t-tests or Analysis of Variance (ANOVA) tests followed by Newman-Keuls *post-hoc* test for multiple comparisons.

## Results

### Systemic eMSC injection at either a single or multiple doses significantly reduces 4T1 primary tumor growth and progression

**Figure 1:**
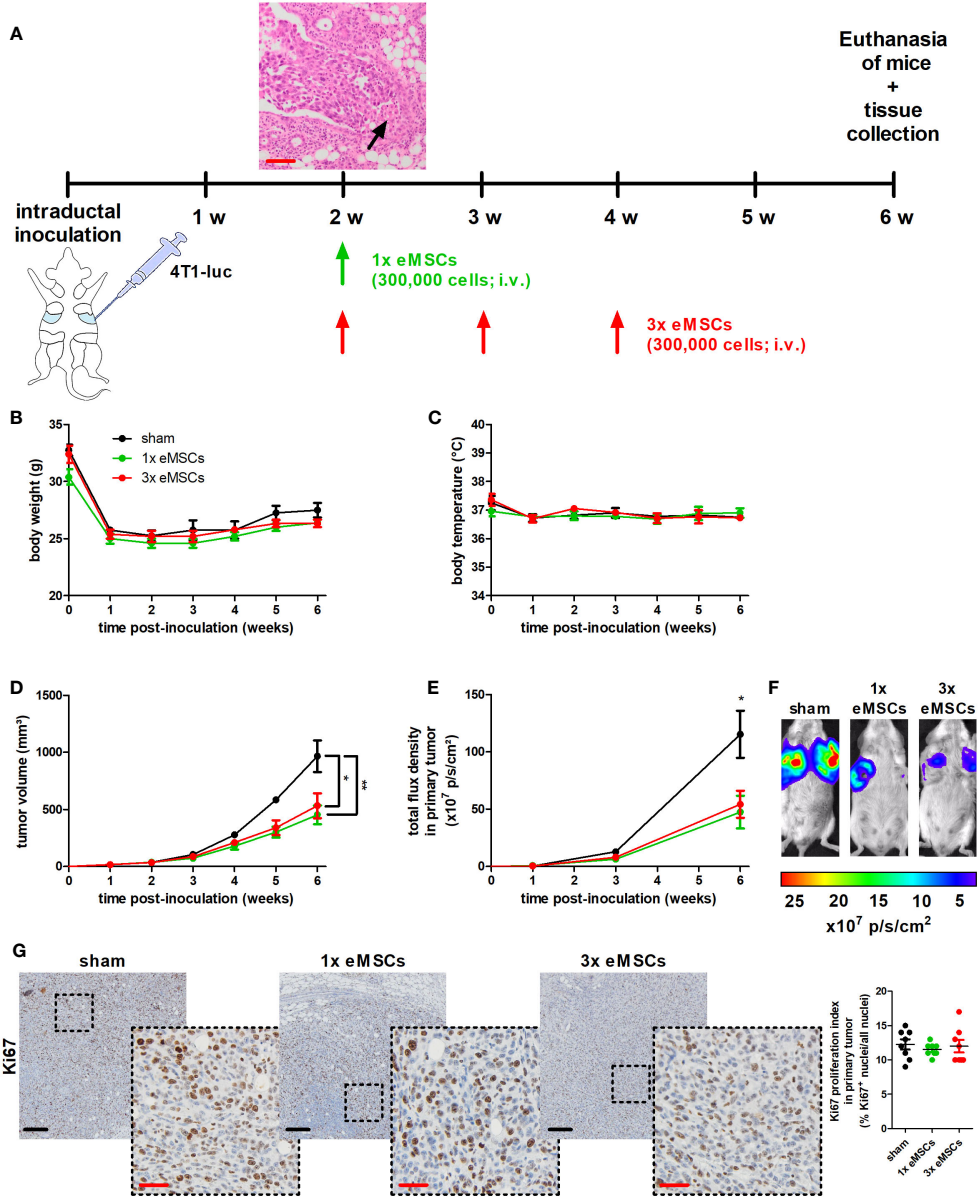
Reduced tumor progression upon eMSC treatment in a 4T1-based intraductal model. **(A)** Experimental timeline with H&E image of primary tumors showing early breakthrough of tumor cells in the mammary fat pad (arrow) at 2 w p.i. and eMSC treatment schedule. **(B, C)** Body weight **(B)** and temperature **(C)** of sham-, 1x eMSC- and 3x eMSC-treated mice across the 6 w study period (n = 4 for the sham group at all time points; n = 5 for the 1x eMSCs group at all time points; n = 5 for the 3x eMSCs group at all time points, except at 5 and 6 w p.i. n = 3). **(D)** Primary tumor volume across the 6 w study period (n = 8 for the sham group at all time points; n = 10 for the 1x eMSCs group at all time points; n = 10 for the 3x eMSCs group at all time points, except at 5 and 6 w p.i. n = 6). **(E)** *In vivo* imaging of bioluminescence in the primary tumor areas (shown as total flux density in p/s/cm²; n = 8 for the sham group at all time points; n = 10 for the 1x eMSCs at all time points; n = 10 for the 3x eMSCs group at all time points, except at 6 w p.i. n = 6). **(F)** Representative images of the primary tumor bioluminescence at 6 w p.i. **(G)** Immunohistochemistry for the cell proliferation marker Ki67 on primary tumor sections at 6 w p.i. (n = 8; 2 tissue slides with 4 images per slide). Dashed inserts show larger magnification of stained tissue. Ki67 proliferation index highlights the ratio of Ki67^+^-stained nuclei to all purple-stained nuclei. Black scale bars = 200 μm, red scale bars = 50 μm. Data are presented as the means +/- SEM. *: *P* < 0.05, **: *P* < 0.01.

Upon their intraductal injection, 4T1 cells already break through the epithelial barrier by 2 w post-inoculation (p.i.), mimicking the progression from ductal carcinoma in situ (DCIS) to invasive carcinoma (IC) observed in TNBC patients (**Figure 1A**). This initial transition was set as the starting point for systemic treatment with a single (1x) or multiple (3x) doses of eMSCs.

First, the homing locations of eMSCs following their tail vein injection were identified through ^99m^Tc-labeling and SPECT/CT tracing. The imaging data showed no uptake of ^99m^Tc-eMSCs for uniformity in 4T1 primary tumors during kinetic measurement (**Supplementary Figures 1A, B**). Instead, the majority of ^99m^Tc-eMSCs migrated to the liver and spleen after 1, 4 and 7 h. Although the lungs were also a homing location for ^99m^Tc-eMSCs, these signals were 8 and 4 times lower at all measured time points than those in liver and spleen, respectively. The signals in the bladder confirmed the urinary excretion of ^99m^Tc. At 22 h after injection, the ^99m^Tc-eMSCs signals in liver, spleen, lungs and bladder decreased due to radioactive decay.

Second, the potential anti-4T1 tumor effects of eMSC treatment were evaluated by monitoring 4T1 tumor progression until 6 w p.i. of the 4T1 cells, i.e. at 4 w after injection of the eMSCs. This treatment did not cause side effects as body weight and temperature of all eMSC-treated 4T1 tumor-bearing mice were comparable to their sham-treated counterparts (DMEM as negative control), with minimal variation across the 6 w study period (**Figures 1B, C**). Additional observations that highlighted the safety of the xenogeneic treatment included the absence of altered behavior (such as auto-mutilation or abnormal reaction to stimuli) and the absence of local skin rash or liver necrosis that could indicate a host-versus-graft response. Lactation allows for efficient and surgery-free intraductal inoculation of the mammary gland, but its cessation is associated with a drop in body weight after 1 w p.i. (**Figure 1B**). Primary tumor volume measurements showed that a single dose of eMSCs already significantly decreased tumor progression by 6 w p.i., compared to the sham treatment (**Figure 1D**). This reduction in tumor growth was also confirmed by *in vivo* imaging at the primary tumor site (**Figures 1E, F**). Based on PCR equine DNA data, eMSCs could not be detected at that time in several organs (including liver, kidneys, spleen, axillary lymph nodes, lungs and primary tumor; **Supplementary Figure 1C**). Comparable Ki67 staining and associated proliferation indexes from eMSC- and sham-treated tumors demonstrated that the tumor growth reduction was not due to a decrease in 4T1 cell proliferation (**Figure 1G**). In addition, the treatment effect of eMSCs could not be further enhanced by increasing the dosing frequency, as weekly dosing of eMSCs from 2 to 4 w p.i. of 4T1 cells induced similar tumor growth reduction and Ki67 proliferation index increase as a single dose at 2 w p.i., also without negatively affecting animal welfare (**Figures 1B-G**).

**Figure 2:**
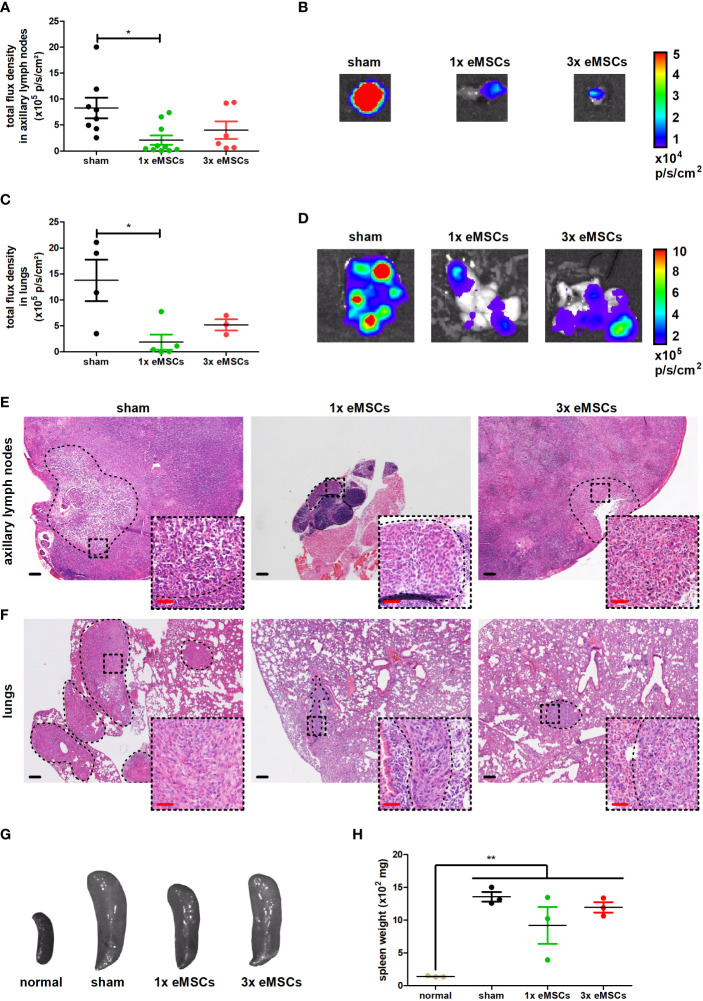
Reduced metastatic progression upon eMSC treatment in a 4T1-based intraductal model. **(A)** Quantification of 4T1-derived bioluminescence in axillary lymph nodes from sham-, 1x eMSC- and 3x eMSC-treated mice at 6 w p.i. based on total flux density (in p/s/cm²) (n = 8 for the sham group; n = 10 for the 1x eMSCs group; n = 6 for the 3x eMSCs group). **(B)** Representative images of bioluminescence in axillary lymph nodes from all groups at 6 w p.i. **(C)** Quantification of 4T1-derived bioluminescence in lungs from all groups at 6 w p.i. based on total flux density (in p/s/cm²) (n = 4 for the sham group; n = 5 for the 1x eMSCs group; n = 3 for the 3x eMSCs group). **(D)** Representative images of bioluminescence signals in lungs from all groups at 6 w p.i. **(E, F)** H&E images of axillary lymph node **(E)** and lung **(F)** metastases in all groups at 6 w p.i. Dashed inserts show larger magnification of highlighted areas. Black scale bars = 200 μm, red scale bars = 50 μm. **(G)** Representative images of the spleen from all groups at 6 w p.i. and from a healthy mouse for comparison. **(H)** Spleen weight from all groups at 6 w p.i. and healthy mice for comparison (n = 3 for all groups). Data are presented as the means +/- SEM. *: *P* < 0.05, **: *P* < 0.01.

*Ex vivo* imaging was used to evaluate metastases and observed a decreased metastatic growth in axillary lymph nodes (**Figures 2A, B**) and lungs (**Figures 2C, D**) with both single and multiple dosing regimens, although only the metastatic decrease with the single dose treatment reached statistical significance. This discrepancy in statistical significance for the 3x eMSCs treatment group due to slightly higher luminescent signals is likely the result of background signals derived from residual circulating 4T1 cells in lymphatic vessels connected to the lymph node and in blood that flows through the lung. We reported that such background signals can similarly hamper metastatic measurements through *ex vivo* imaging in the liver (20), necessitating confirmation of metastatic reduction on tissue slides. H&E histology confirmed the presence of smaller and fewer metastases in axillary lymph nodes and lungs in eMSC- compared to sham-treated mice (**Figures 2E, F**).

Splenomegaly has been associated with tumor progression and leukemoid reactions in the 4T1 model for TNBC, as reviewed by our group (21). The weight of the spleen in all tumor-bearing groups was significantly increased compared to a healthy spleen, and eMSC treatment did not reduce the spleen weight despite the decrease in both primary tumor growth and metastases in eMSC-treated mice (**Figures 2G, H**).

In order to evaluate whether the eMSC-mediated anti-4T1 tumor effect could be replicated by other equine stem cell types, eEpSCs were intravenously injected (**Supplementary Figure 2A**). They established a comparable reduction in primary tumor growth at 6 w p.i. following either 1 or 3 doses, without toxicity or change in tumor cell proliferation based on Ki67 staining (**Supplementary Figures 2B-G**). Further in line with eMSCs, single and multiple eEpSC treatment doses provided comparable significantly reduced axillary lymph node and lung metastases without causing reduced splenomegaly (**Supplementary Figures 3A-H**).

### Systemic eMSC-induced reduction in 4T1 primary tumor growth and progression is associated with T-cell infiltration and local increase in inflammatory cytokines

**Figure 3:**
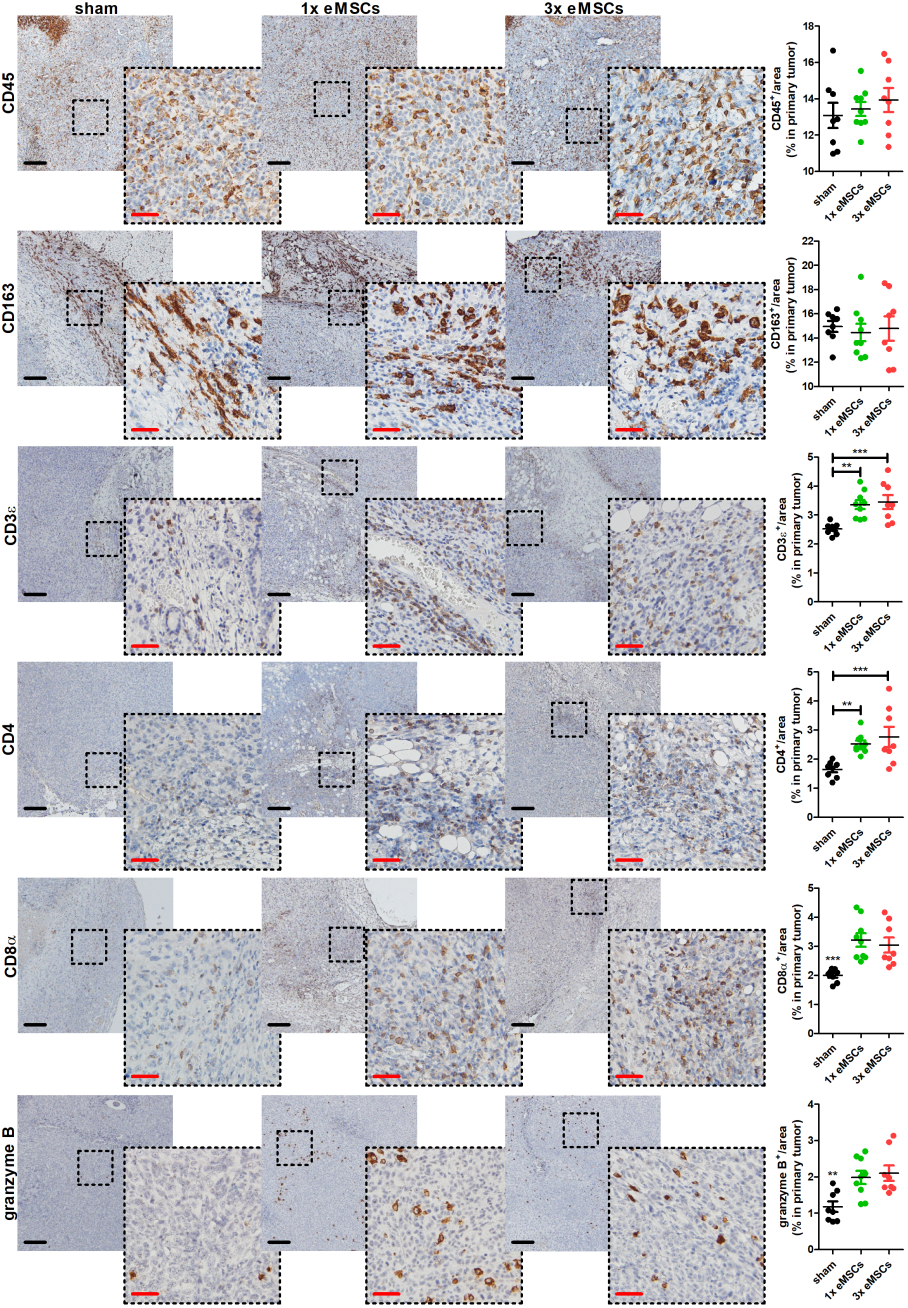
Increased T-cell infiltration and activation in primary tumors upon eMSC treatment in a 4T1-based intraductal model. Immunohistochemistry for the leukocyte marker CD45, the M2 TAM marker CD163, the T-cell marker CD3ϵ, the specific T-cell subtype markers CD4 and CD8α, and the lymphocytic activation marker granzyme B on primary tumor sections from sham-, 1x eMSC- and 3x eMSC-treated mice at 6 w p.i. (n = 8 for all groups; 2 tissue slides with 4 images per slide). Dashed inserts highlight stained tissue at a larger magnification. Black scale bars = 200 µm, red scale bars = 50 µm. Data are presented as the means +/- SEM. **: *P* < 0.01, ***: *P* < 0.001.

To investigate immunological changes that accompany the eMSC-mediated disease reduction, tumor-associated cyto-/chemokine profiles were analyzed at 6 w p.i. in target organs, including primary tumor, axillary lymph nodes, lungs and spleen. Screening of 10 cyto-/chemokines in these tissues of eMSC- compared to sham-treated mice showed that eMSC treatment increases several pro-inflammatory/anti-tumorigenic cyto-/chemokines in primary tumors treated with one or multiple eMSC doses compared to sham-treated controls. However, only the increase in G-CSF levels upon 1x and 3x eMSC treatment compared to sham treatment, IL-6 levels upon 1x eMSC treatment compared to sham treatment, and MIP-2 levels upon 3x eMSC compared to 1x eMSC and sham treatment was significant (**Supplementary Figure 4A**). In contrast, cytokine profile changes induced by eMSC treatment in all other investigated organs were not statistically significant (**Supplementary Figures 4B-D**). Overall, immune cell abundance did not significantly change upon treatment with either a single or multiple eMSC doses based on CD45 immunostaining on primary tumor tissue at 6 w p.i. (**Figure 3**). Similar findings were obtained for anti-inflammatory M2 tumor-associated macrophages (TAMs) at the tumor margin based on CD163 staining (**Figure 3**). On the other hand, T-cells significantly increased in eMSC-treated primary tumors based on CD3ϵ staining, further specified as an increase in both CD4^+^ and CD8α^+^ T-cells (**Figure 3**). Moreover, staining for granzyme B, as marker for T-cell and NK(-T) cell activation, identified that stimulation of cytotoxicity accompanied the increased T-cell influx in eMSC- compared to sham-treated mice (**Figure 3**).

Upon treatment with single or multiple doses of eEpSCs, similar to eMSC treatment, several pro-inflammatory cyto-/chemokines were increased in primary tumors compared to sham-treated controls, although only the increase in MCP-1 levels upon 1x eEpSC treatment was significant (**Supplementary Figure 5A**). Also similar to eMSC treatment, cytokine profiles were not significantly changed by eEpSC treatment in other investigated organs, except for a significant increase in splenic MIP-2 levels upon 1x eEpSC treatment (**Supplementary Figures 5B-D**). The numbers of CD45^+^ pan-leukocytes and CD163^+^ M2 TAMs were again unaltered, whereas CD3ϵ^+^ and more specifically the CD4^+^ and CD8α^+^ T-cells along with the cytotoxic marker granzyme B again significantly increased compared to sham-treated 4T1 primary tumors based on immunohistochemistry (**Supplementary Figure 6**).

### Systemic eMSC treatment after splenectomy still enhances 4T1 primary tumor T-cell infiltration

**Figure 4:**
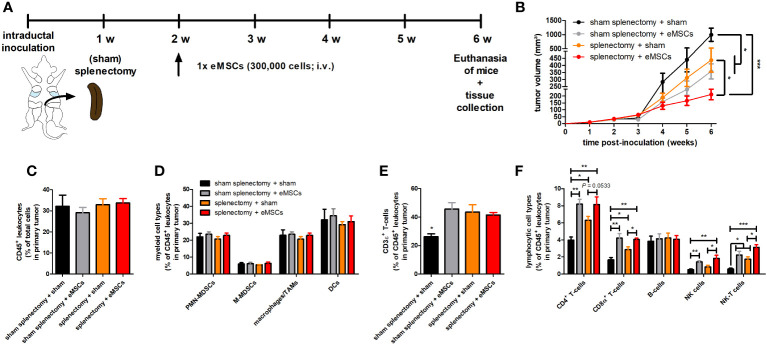
Splenectomy does not impact eMSC-mediated primary tumor reduction and T-cell infiltration in a 4T1-based intraductal model. **(A)** Experimental timeline with (sham) splenectomy at 1 w p.i. and eMSC treatment schedule indicated. **(B)** Primary tumor volume across the 6 w study period (n = 10 for all groups at all time points, except for the sham splenectomy + sham group at 4, 5 and 6 w p.i. n = 8). **(C–F)** Flow cytometric immunophenotyping of primary tumors from all groups at 6 w p.i. (n = 4 for the sham splenectomy + sham group; n = 5 for all other groups). **(C)** Percentage of CD45^+^ leukocytes within the total cell suspension. **(D)** Percentage of myeloid cell types (including PMN-MDSCs, M-MDSCs, macrophages/TAMs and DCs) within the CD45^+^ leukocyte population. **(E)** Percentage of CD3ϵ^+^ T-cells within the CD45^+^ leukocyte population. **(F)** Percentage of lymphocytic cell types (including CD4^+^ and CD8α^+^ T-cells, B-cells, NK and NK-T cells) within the CD45^+^ leukocyte population. Data are presented as the means +/- SEM. *: *P* < 0.05, **: *P* < 0.01, ***: *P* < 0.001.

Since eMSCs were primarily trapped inside the spleen upon their systemic injection, it was investigated whether the spleen plays a role in the observed primary tumor immune effects and eMSC treatment effect. Tumor-bearing mice underwent either splenectomy or sham splenectomy at 1 w p.i. of 4T1 cells followed by either eMSC or sham (DMEM) treatment at 2 w p.i., and tumor progression was monitored until 6 w p.i. (**Figure 4A**).

Body weight and temperature of all tumor-bearing mice showed minimal variation across the 6 w study period, indicating that neither of the evaluated treatments nor the (sham) splenectomy negatively impacted animal health (**Supplementary Figures 7A, B**). Based on primary tumor volume (**Figure 4B**) and *in vivo* bioluminescence (**Supplementary Figures 7C, D**), splenectomy + sham treatment significantly reduced tumor progression compared to sham splenectomy + sham treatment in tumor-bearing mice. This result identifies the spleen as tumor-stimulating organ, probably due to its production of immunosuppressive myeloid cells (21). Moreover, eMSC treatment provided a significant additional reduction in primary tumor volume (**Figure 4B**) and bioluminescence imaging at 6 w p.i. in the splenectomized tumor-bearing mice (**Supplementary Figures 7C, D**).

The 4T1-derived bioluminescence was also significantly decreased in metastatic organs i.e. axillary lymph nodes and lungs following eMSC treatment, and prior splenectomy did not affect this reduction (**Supplementary Figures 7E-H**). Although the splenectomy + eMSC treatment showed an additional reduction in all metastatic signals compared to splenectomy + sham treatment, this metastatic decrease was only statistically significant in the lungs. Moreover, the similar increase in spleen weight in the sham-splenectomized mice treated with either eMSCs or sham (**Supplementary Figure 7I**), corroborated previous findings (**Figures 2G, H**). It was investigated whether this splenomegaly was also associated with similar percentages of splenic immune cells by performing flow cytometric immunophenotyping (**Supplementary Figure 8**). Overall, percentages of total splenic CD45^+^ leukocytes and also more specific splenic innate immune cells (including CD45^+^CD11b^+^Ly6C^int^Ly6G^+^ polymorphonuclear (PMN)-myeloid-derived suppressor cells (MDSCs), CD45^+^CD11b^+^Ly6C^hi^Ly6G^-^ monocytic (M)-MDSCs, CD45^+^CD11b^+^F4/80^+^ TAMs and CD45^+^CD11c^+^ dendritic cells (DCs)) (**Supplementary Figures 9A, B**) as well as splenic adaptive immune cells (including CD3ϵ^+^ T-cells and more specific CD45^+^CD3ϵ^+^CD4^+^CD8α^-^ and CD45^+^CD3ϵ^+^CD4^-^CD8α^+^ T-cell subpopulations, CD45^+^CD19^+^ B-cells, CD45^+^CD3ϵ^-^NKp46^+^ natural killer (NK) cells and CD45^+^CD3ϵ^+^NKp46^+^ NK-T cells) (**Supplementary Figures 9C, D**) did not significantly differ between all treatments.

Complementary immunophenotyping on primary tumors showed that (sham) splenectomy followed by eMSC treatment did not affect the overall percentage of CD45^+^ leukocytes, nor that of innate immune cells (i.e. PMN- and M-MDSCs, TAMs and DCs) (**Figures 4C, D**). In marked contrast, primary tumors receiving eMSC treatment after sham splenectomy showed a significant increase in percentages of CD3ϵ^+^ T-cells and specific CD4^+^ and CD8α^+^ T-cell subpopulations as well as in percentages of NK and NK-T cells but not in that of B-cells compared to sham treatment (**Figures 4E, F**). A significant increase in percentages of CD3ϵ^+^ T-cells and both T-cell subpopulations as well as NK-T cells was also observed in sham-treated splenectomized mice (**Figures 4E, F**), indicative for enhanced T-cell-mediated anti-4T1 tumor responses upon splenectomy. However, eMSC treatment further enhanced the T-cell-mediated adaptive immunity and the percentage of NK(-T) cells in splenectomized mice (**Figure 4F**). Significantly increased primary tumor staining for CD4, CD8α and NCR-1 as NK(-T) cell marker upon eMSC compared to sham treatment in all operated mice corroborated the flow cytometry results (**Supplementary Figure 10**). Furthermore, granzyme B staining in sham splenectomized mice was significantly enhanced by eMSC compared to sham treatment (**Suppl. Figure 10**). Splenectomy also significantly increased granzyme B staining compared to sham splenectomy, and the eMSC treatment further enhanced these staining levels (**Supplementary Figure 10**).

### The eMSC treatment effect is hampered by CD4^+^ or CD8α^+^ T-cell depletion

**Figure 5:**
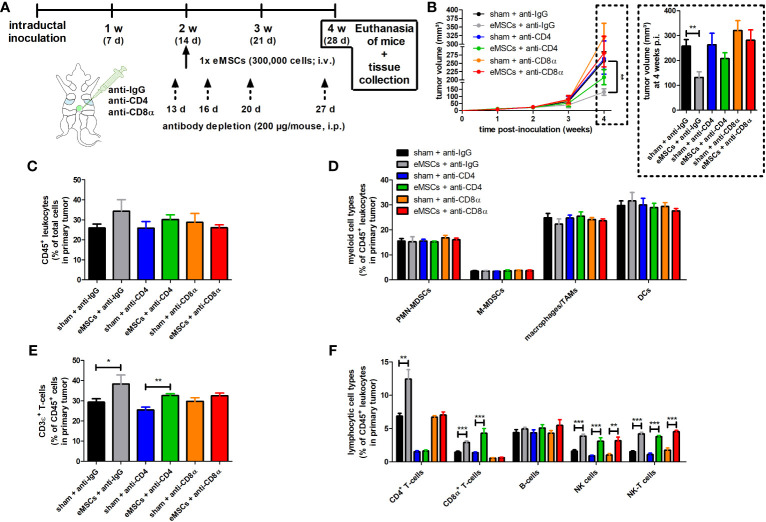
Depletion of CD4^+^ and CD8α^+^ T-cells hampers eMSC treatment effect in a 4T1-based intraductal model. **(A)** Experimental timeline with anti-IgG control, -CD4 and -CD8α depletion starting at 13 d p.i. and eMSC treatment schedule indicated. **(B)** Primary tumor volume monitoring across the 4 w study period (n = 10 for all groups at all time points, except for the sham + anti-CD8α group at 3 and 4 w p.i. n = 8). **(C-F)** Flow cytometric immunophenotyping of primary tumors from all groups at 4 w p.i. (n = 4 for the sham + anti-CD8α group; n = 5 for all other groups). **(C)** Percentage of CD45^+^ leukocytes within the total cell suspension. **(D)** Percentage of myeloid cell types (including PMN-MDSCs, M-MDSCs, macrophages/TAMs and DCs) within the CD45^+^ leukocyte population. **(E)** Percentage of CD3ϵ^+^ T-cells within the CD45^+^ leukocyte population. **(F)** Percentage of lymphocytic cell types (including CD4^+^ and CD8α^+^ T-cells, B-cells, NK and NK-T cells) within the CD45^+^ leukocyte population. Data are presented as the means +/- SEM. *: *P* < 0.05, **: *P* < 0.01, ***: *P* < 0.001.

To assess whether CD4^+^ and CD8α^+^ T-cells are drivers in the eMSC treatment effect, these subpopulations were each separately depleted in 4T1 tumor-bearing mice through specific antibodies followed by eMSC or sham-treatment (**Figure 5A**). Due to antibody toxicity, monitoring of tumor progression was limited to 4 w p.i. of 4T1 cells and 2 w after eMSC injection. Body weight and temperature of all treated tumor-bearing mice showed minimal variation across the 4 w study period (**Supplementary Figures 11A, B**). In the isotype control group, eMSCs significantly reduced tumor volumes compared to sham treatment at 4 w p.i. (**Figure 5B**). Depletion showed that CD4^+^ and, even to a higher extent, CD8α^+^ T-cells were necessary for eMSC-mediated growth reduction. Although a trend towards increased tumor growth was visible with anti-CD8α both upon sham and eMSC treatment, statistical analysis showed no significant differences in tumor volume compared to anti-IgG control (**Figure 5B**). These data indicate that T-cells are not able to effectively combat disease progression in 4T1 tumor-bearing mice. Again corroborating previous findings (**Figures 2G, H**), splenomegaly was not affected by either eMSC or sham treatment (**Supplementary Figure 11C**). In line with the primary tumor growth, a trend towards increased spleen weight was detectable with anti-CD8α both upon sham and eMSC treatment, but no statistically significant difference was found compared to anti-IgG control spleen weights (**Supplementary Figure 11C**).

Similar to 4T1 primary tumors in (sham) splenectomized mice receiving eMSC or sham treatment, immunophenotyping showed that percentages of CD45^+^ leukocytes and innate immune cells (i.e. PMN- and M-MDSCs, TAMs and DCs) were not significantly altered in any of the groups (**Figures 5C, D**). In contrast, CD4^+^ and CD8α^+^ T-cell depletion showed differential effects on the total CD3ϵ^+^ T-cell percentage, with anti-CD4 depletion not affecting and anti-CD8α depletion preventing an eMSC-mediated T-cell percentage increase (**Figure 5E**). This indicates that CD8α^+^ T-cells are the most important T-cell subset for the eMSC-mediated CD3ϵ^+^ adaptive immunity increase. As a control for successful depletion, CD4^+^ and CD8α^+^ T-cells were not detectable in primary tumors of mice upon anti-CD4 and anti-CD8α depletion, respectively (**Figure 5F**), and this depletion was corroborated in blood samples (**Supplementary Figure 12**). In eMSC-treated mice, primary tumor CD8α^+^ T-cell percentages significantly increased, but, similarly to total CD3ϵ^+^ T-cell percentages, CD4^+^ T-cell depletion did not affect this increase (**Figure 5F**). In marked contrast, CD8α depletion inhibited the significant eMSC-mediated increase in primary tumor CD4^+^ T-cell percentages (**Figure 5F**). The percentages of NK(-T) cells also significantly increased by eMSC treatment and neither isotype control, CD4^+^ or CD8α^+^ T-cell depletion impacted this increase (**Figure 5F**). Immunophenotyping was corroborated by the unaffected eMSC-mediated increase in CD3ϵ and CD8α staining upon anti-CD4 depletion and the inhibited eMSC-mediated increase in CD4 staining upon anti-CD8α depletion in primary tumors (**Supplementary Figure 13**). Treatment with eMSCs also significantly increased NK(-T) cells as confirmed through NCR-1 staining (**Supplementary Figure 13**). Due to the unaffected increase in NK(-T) cells, granzyme B staining remained significantly increased upon eMSC treatment, and neither CD4^+^ or CD8α^+^ T-cell depletion had an impact on this increase (**Supplementary Figure 13**).

## Discussion

This is the first report to evaluate eMSCs derived from horse peripheral blood as xenogeneic cell type for anti-TNBC treatment. Our preclinical results show that i.v. injection of eMSCs in 4T1 tumor-bearing mice safely and significantly reduce primary tumor and metastatic growth. The safety of eMSCs has already been shown in several non-rodent species with relevance for veterinary medicine, including cats (22) and dogs (18, 23–25). Although the spleen is one of the major entrapment sites for eMSCs, its removal did not significantly impact their treatment effect. Moreover, also eEpSCs (as alternative equine stem cell type) provide similar disease reduction compared to eMSCs, identifying that the type of equine stem cells does not impact *in vivo* treatment. Mechanistically, we show that both eMSC and eEpSC treatment induces T- and NK(-T) cell-mediated responses in primary tumors, which we hypothesize is related to xenogeneic cell rejection (26). The latter process is initialized through the recognition of xeno-antigens by antigen-presenting cells (APCs), including macrophages and dendritic cells, at the homing locations of the xenogeneic cells. The remarkable observation that splenic removal did not hamper the eMSC-mediated anti-4T1 tumor effects, strongly suggests that recognition and capturing of the presumed equine xeno-antigens occurs independently across different homing locations with equal potential to evoke anti-tumorigenic effects. In accordance with the generally accepted cancer-immunity cycle concept (27), these APCs subsequently drive the anti-tumorigenic responses by travelling towards the lymph nodes where they present the xeno-antigens to residing CD8α^+^ T-cells, priming and activating effector T-cell responses. The activated CD8α^+^ T-cells are subsequently released from the lymph nodes and travel the bloodstream in search for target cells. They eventually infiltrate the primary tumor or its metastatic sites to perform off-target cytotoxic activity towards tumor cells. Based on this proposed theory, xenogeneic equine stem cells could have the potential to revive anti-tumor immunity and change immune-deprived ‘cold’ into immune-activated ‘hot’ tumors (28) without having to directly reach the primary tumor site. Of relevance, CD4^+^ T-cells may also be involved in this process, further stimulating anti-tumorigenic effector T-cell responses as well as activating NK(-T) cells to perform direct (tumor) cell killing (26, 29). The observation that the spleen size does not decrease despite reduced 4T1 primary tumor and metastatic growth is probably also related to the xenogeneic cell rejection process. Indeed, neutrophils and PMN-MDSCs as neutrophil precursors have been reported to be one of the first immune cell types to reach the homing locations of xenogeneic cells following their systemic injection (30). Similarly to a classic innate immune response to pathogens, these neutrophils initiate cell-mediated immunity by attracting monocytes/macrophages for subsequent T-cell activation, thereby potentially increasing spleen size.

Using T-cell depletion experiments, we verified that xenogeneic eMSCs critically rely on the increase of CD4^+^ and CD8α^+^ T-cell infiltration in 4T1 primary tumors and the concomitant increase in the lymphocytic activation marker granzyme B for their treatment effect, again likely related to xenogeneic cell rejection. As a drawback of our study, we did not verify the necessity of CD4^+^ and CD8α^+^ T-cell infiltration and activation for eEpSC treatment effects through T-cell depletion experiments. Indeed, this should be ideally investigated to identify T-cell dependency for anti-cancer efficacy as a general characteristic across different equine stem cell types. Although NK(-T) cells are an important bystander population that significantly increase granzyme B positivity in eMSC-treated primary tumors, even upon depletion of CD4^+^ and CD8α^+^ T-cells, we here show that they are unable to provide significant disease reduction without the help of T-cells.

Besides their potential anti-tumorigenicity through xenogeneic rejection responses, several studies also identified that MSCs can exert anti-tumor activity through paracrine effects and predominantly via secretion of extracellular vesicles (31, 32). Therefore, MSCs have been introduced as carrier cells to deliver anti-cancer agents such as doxorubicin (33, 34), but also oncolytic viruses that stimulate pro-apoptotic pathways (35, 36). A noteworthy advantage of MSCs is that they are able to secrete transduced immunostimulatory proteins for a long time, which is especially interesting when using short-lived cytokines such as IFN-β (37). In line with this, engineered MSCs were recently designed to deliver IL-2 to tumor-infiltrating CD8^+^ T-cells and were thereby able to expand this immune population, inducing systemic anti-tumor immunity and alleviating ICB resistance (38). It may be hypothesized that the evoked anti-4T1 tumor immunity based on xenogeneic cell rejection can be further invigorated by loading eMSCs with additional immunostimulatory cytokines, but this strategy requires rigorous cellular manipulation (39).

It is a curious finding that both 1 and 3 doses of eMSCs (and eEpSCs) cause similar reduction in disease progression and increase in immune responses. One cause for the observed absence of a dose effect may be the rapid exhaustion of the host immune system, indicating that a single dose of xenogeneic eMSCs could already strongly stimulate the immune system and result in dysfunctional T-cells. If this is the case, such exhaustion would preclude the intended additional T-cell activation by repeated eMSC dosing. Alternatively, immunosuppressive myeloid cells can also hamper T-cell stimulation and thus the effect of repeated treatment (40). Indeed, a salient finding is that MDSCs based on flow cytometrical analysis and immunosuppressive CD163^+^ (i.e. M2-polarized) TAMs based on immunohistochemistry are not affected by eMSC treatment and remain abundant in the TME, even upon tumor growth reduction. Therefore, this unaffected presence of MDSCs and macrophages upon single and multiple eMSC doses potentially further inhibits the intended additional T-cell infiltration in the TME as well as the required T-cell activation. The dysfunctional or exhausted T-cell state is typically regulated by programmed death (PD)-1/PD-ligand (L)1 signaling, in which PD-1 expressed by the T-cell is bound by PD-L1 present on the mammary tumor cells as well as the MDSCs and macrophages in the TME (40, 41). Given the importance of active T-cells in primary tumor reduction, these immune checkpoint proteins are intensively investigated as targets for cancer treatment (40, 42). While promising results with ICBs (i.e. antibodies against PD-1 and PD-L1) have been obtained (17, 43, 44), the clinical failure in TNBC patients remains high due to tolerance effects (45, 46). Future experiments are therefore warranted to investigate PD-1/PD-L1 expression in the TME following eMSC dosing. As such increased expression has been linked to T-cell exhaustion, it could substantiate why repeated eMSC treatment does not provide additional disease reduction. Based on our current findings, we therefore suggest it may be relevant to evaluate whether a combination of eMSCs with ICB treatment is superior in comparison with both these single treatments, potentiating additional T-cell stimulation and disease reduction upon repeated eMSC dosing.

Also of interest, the disease reduction and absence of tumorigenic effects associated with the use of eMSCs can be postulated as contrasting to hMSCs, which have been described as oncogenic (47–50) and rather aggravate tumor growth by mediating immunosuppression (51). Yet, there may be several possible reasons for this discrepancy in tumorigenicity between eMSCs and hMSCs. Firstly, i.v. injected eMSCs are rapidly cleared (already after 22 h based on SPECT/CT biodistribution, although the lack of intermediate tracking analysis of eMSCs during the middle of treatment should be highlighted as a limitation) and do not show homing to the primary mammary tumor in mice, whereas hMSCs have been reported to consistently home to the mouse mammary tumor site and remain detectable for several days (52), providing the correct environment and enough time to exert undesired tumorigenic effects. Secondly, there may be species differences between the hMSC and eMSC secretome that determine their difference in tumorigenicity *in vivo*. Although the characterization of the eMSC secretome has only just been started, a recent study reported at first the isolation of exosomes from eMSCs with similar positivity for CD9 and CD63 as human-derived exosomes (53). However, the cargo of these exosomes and other secreted proteins needs to be further investigated as these could further explain differences with oncogenic hMSCs and also potentially the observed anti-cancer effects in our current study. In this regard, a less controversial alternative for systemic anti-cancer therapy could be tested through the use of acellular eMSC-derived extracellular vesicles or exosomes. A recent study already showed that these extracellular vesicles are capable of decreasing inflammation similarly to the eMSCs from which they were derived (54). Thirdly, tumorigenicity and related adverse events are highly dependent on the purity of the MSCs. Most reports use heterogenous hMSC populations and fail to preselect tumor-reactive hMSCs (55). The eMSCs used in our study are thoroughly characterized in a GMP facility based on previously published protocols to ensure a homogenous and highly pure eMSC population (13, 14). Only eMSC batches that pass these acceptance criteria are used for systemic injection and downstream analyses, providing necessary quality control for future clinical application. Besides providing a plausible explanation for the difference in tumorigenicity between eMSCs and hMSCs, these arguments can also clarify a potential difference in immunomodulation between equine stem cells and differentiated xenogeneic cell types. Indeed, fast clearance following systemic administration (8), the secretome (56–59) and the use of a more homogenous cellular population for injection of equine stem cells compared to other xenogeneic cells may provide subtle differences in (durable) immunostimulation that can have an important impact on preventing disease recurrence. Future studies are therefore warranted to investigate the immunological effects of equine stem cells in a side-by-side comparison with more differentiated xenogeneic cell types, potentially also from other species.

## Data availability statement

The raw data supporting the conclusions of this article will be made available by the authors, without undue reservation.

## Ethics statement

The animal study was approved by the ethical committee (EC) of the Faculty of Veterinary Medicine, Ghent University. The study was conducted in accordance with the local legislation and institutional requirements.

## Author contributions

JS, GP, JHS, and EM concepted and designed the study. JS, KD, ND, and HDR acquired the data. JS and KD analyzed and interpreted the data. JS drafted the manuscript. GP, ND, HDR, NS, JHS, and EM revised the manuscript. All authors contributed to the article and approved the submitted version.
