# Supplementary material for: Xenogeneic equine stem cells activate anti-tumor adaptive immunity in a 4T1-based intraductal mouse model for triple-negative breast cancer: proof-of-principle
Source: Front Immunol. 2023 Oct 20;14:1252374. doi: 10.3389/fimmu.2023.1252374 (PMC10623058; doi:10.3389/fimmu.2023.1252374)
Supplement: Supplementary file 1 [file DataSheet_1.pdf]

Supplementary Material

Xenogeneic equine stem cells activate anti-tumor adaptive immunity in a 4T1-based intraductal mouse model for triple-negative breast cancer: proof-of-principle

Jonas Steenbrugge[1,2,*], Glenn Pauwelyn[3], Kristel Demeyere[1], Nausikaa Devriendt[4], Hilde de Rooster[2,4], Niek N. Sanders[2,5], Jan H. Spaas[6,7,†] and Evelyne Meyer[1,2,†]

[1]Laboratory of Biochemistry, Department of Veterinary and Biosciences, Faculty of Veterinary Medicine, Ghent University, Merelbeke, Belgium

[2]Cancer Research Institute Ghent (CRIG), Ghent, Belgium

[3]Boehringer Ingelheim Veterinary Medicine Belgium, Evergem, Belgium

[4]Small Animal Department, Faculty of Veterinary Medicine, Ghent University, Merelbeke, Belgium.

[5]Laboratory of Gene Therapy, Department of Veterinary and Biosciences, Faculty of Veterinary Medicine, Ghent University, Merelbeke, Belgium

[6]Department of Morphology, Imaging, Orthopedics, Rehabilitation and Nutrition, Faculty of Veterinary Medicine, Ghent University, Merelbeke, Belgium.

[7]Boehringer-Ingelheim Animal Health USA, Athens, GA, United States.

[*]Corresponding author: Jonas Steenbrugge; phone number: +3292647356; fax number: +3292647497; e-mail: Jonas.Steenbrugge@ugent.be

[†]Shared last authorship

Supplementary Fig. 1

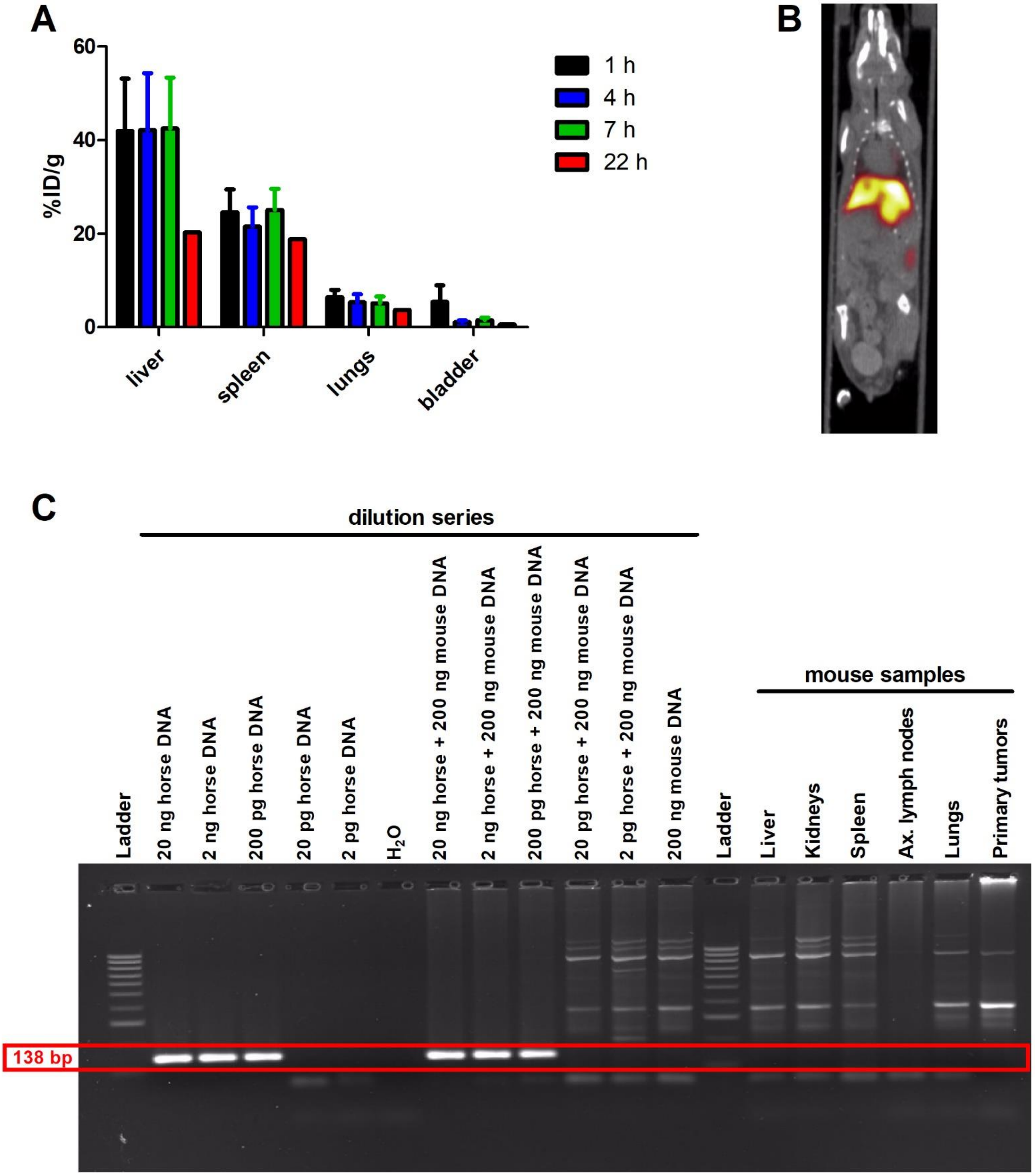

Supplementary Figure 1. SPECT/CT biodistribution of ^{99m}Tc-eMSCs and eMSC-derived horse DNA detection in a 4T1-based intraductal model

(**A**) SPECT/CT biodistribution in liver, spleen, lungs and bladder shown as % injected dose per gram tissue (%ID/g) across 4 different time points (n = 3 for 1, 4 and 7 h; n = 1 for 22 h after ^{99m}Tc-eMSC administration). No signal was detected in primary tumors. (**B**) Representative image of SPECT/CT from a 4T1 tumor-bearing mouse at 7 h after ^{99m}Tc-eMSC administration. (**C**) Gel with PCR-amplified dilution series of horse DNA as control (specific horse DNA band detectable at 138 bp), and absence of horse DNA in liver, kidneys, spleen, axillary lymph nodes, lungs and primary tumors at 6 w p.i. as a confirmation for the absence of eMSCs. Data are presented as the means +/- SEM.

Supplementary Fig. 2

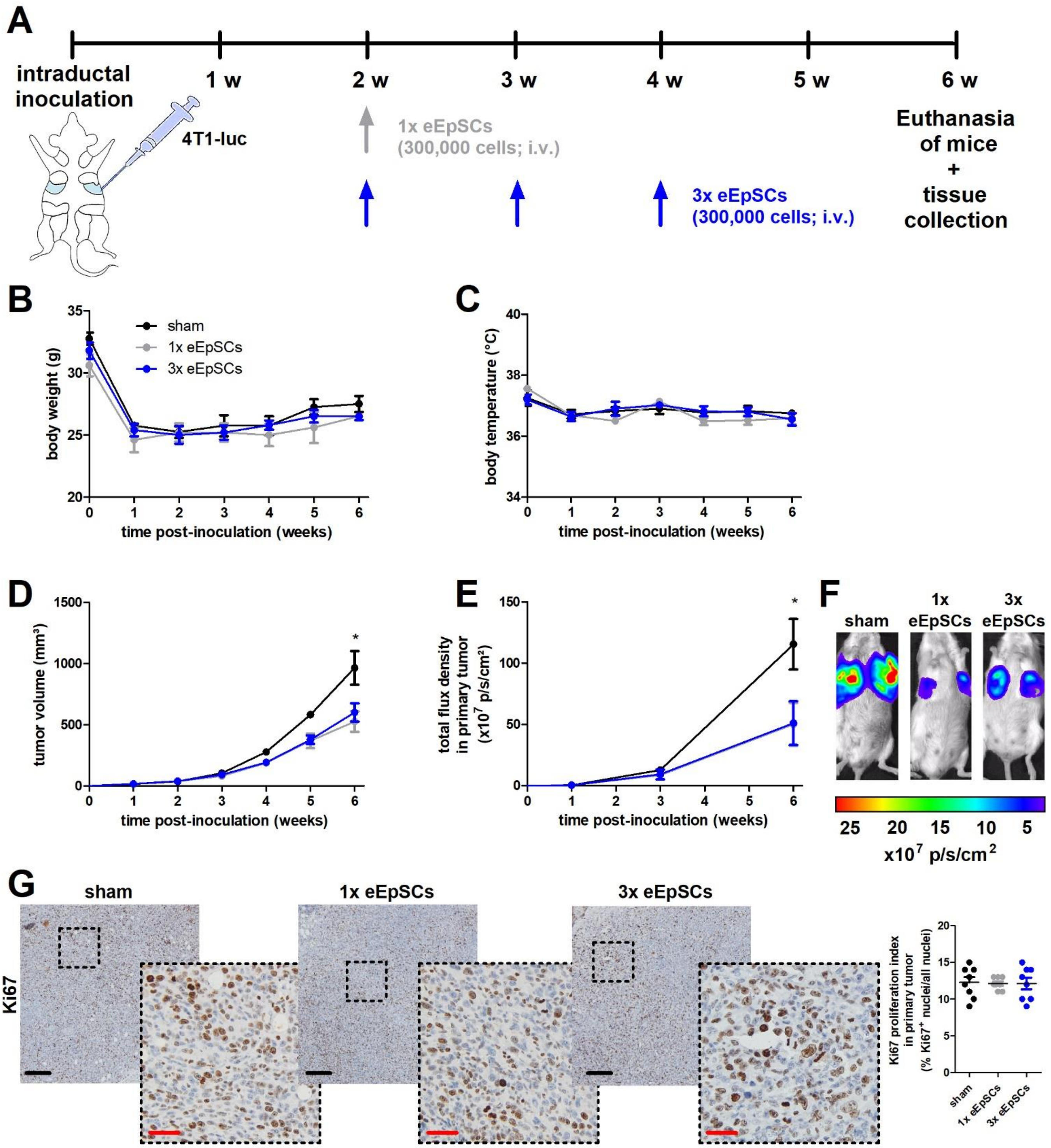

Supplementary Figure 2. Reduced tumor progression upon eEpSC treatment in a 4T1-based intraductal model

(**A**) Experimental timeline with eEpSC treatment schedule. (**B, C**) Body weight (**B**) and temperature (**C**) of sham-, 1x eEpSC- and 3x eEpSC-treated mice across the 6 w study period (n = 4 for the sham group at all time points; n = 5 for the 1x eEpSCs group at all time points, except at 6 w p.i. n = 4; n = 5 for the 3x eEpSCs group at all time points, except at 5 and 6 w p.i. n = 4). (**D**) Primary tumor volume across the 6 w study period (n = 8 for the sham group at all time points; n = 10 for the 1x eEpSCs group at all time points, except at 6 w p.i. n = 8; n = 10 for the 3x eEpSCs group at all time points, except at 5 and 6 w p.i. n = 8). (**E**) *In vivo* imaging of bioluminescence in the primary tumor areas (shown as total flux density in p/s/cm^2; n = 8 for the sham group at all time points; n = 10 for the 1x eEpSCs and 3x eEpSCs group at all time points, except at 6 w p.i. n = 8). (**F**) Representative images of the primary tumor bioluminescence at 6 w p.i. (**G**) Immunohistochemistry for the cell proliferation marker Ki67 on primary tumor sections at 6 w p.i. (n = 8 for all groups; 2 tissue slides with 4 images per slide). Dashed inserts show larger magnification of stained tissue. Ki67 proliferation index highlights the ratio of Ki67$^+$-stained nuclei to all purple-stained nuclei. Black scale bars = 200 µm, red scale bars = 50 µm. Data are presented as the means +/- SEM. *: $P < 0.05$.

Supplementary Fig. 3

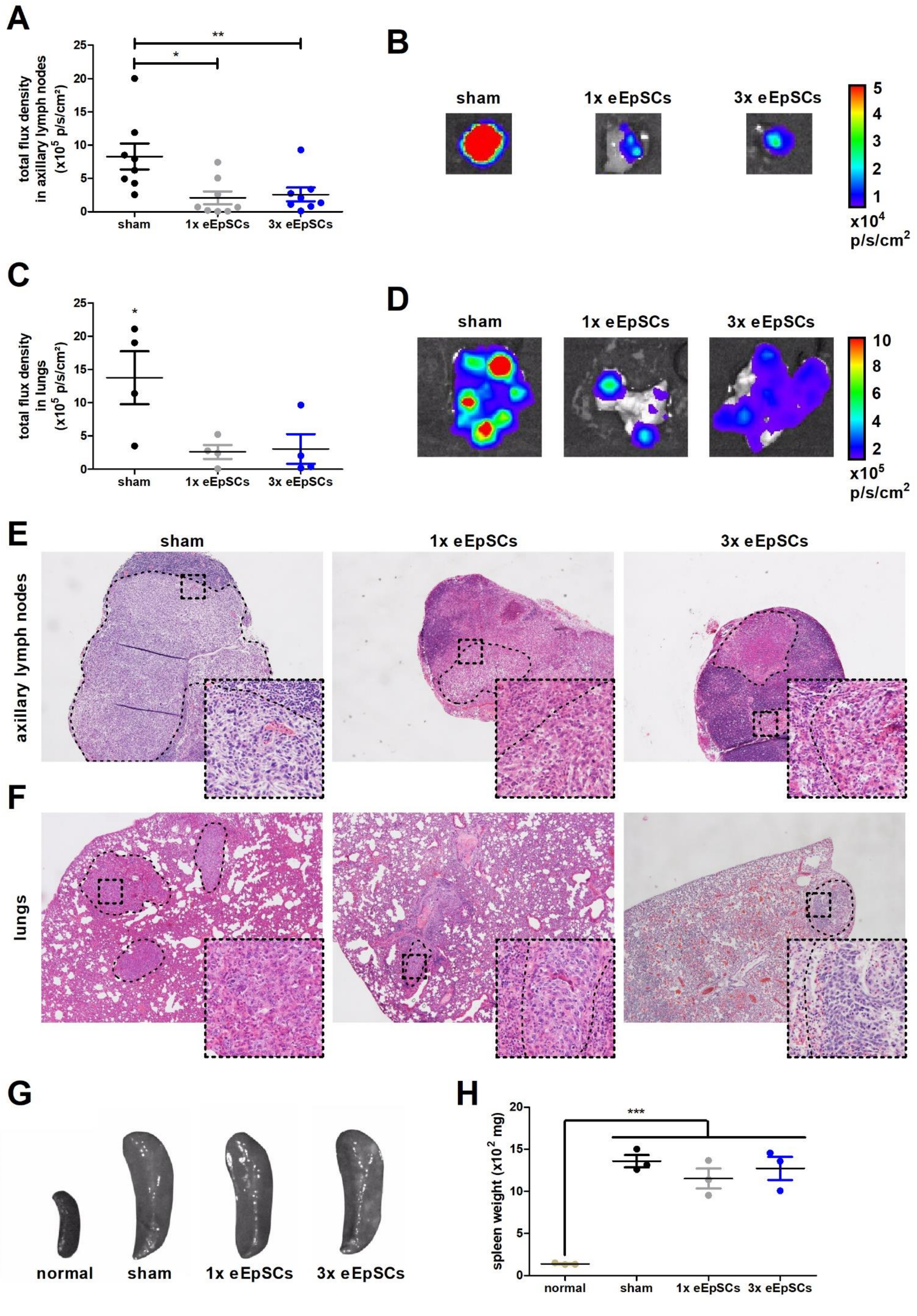

Supplementary Figure 3. Reduced metastatic progression upon eEpSC treatment in a 4T1-based intraductal model

(**A**) Quantification of 4T1-derived bioluminescence in axillary lymph nodes from sham-, 1x eEpSC- and 3x eEpSC-treated mice at 6 w p.i. based on total flux density (in p/s/cm²) (n = 8 for all groups). (**B**) Representative images of bioluminescence in axillary lymph nodes from all groups at 6 w p.i. (**C**) Quantification of 4T1-derived bioluminescence in lungs from all groups at 6 w p.i. based on total flux density (in p/s/cm²) (n = 4 for all groups). (**D**) Representative images of bioluminescence in lungs from all groups at 6 w p.i.. (**E, F**) H&E images of axillary lymph node (**E**) and lung (**F**) metastases in all groups at 6 w p.i. Dashed inserts show larger magnification of highlighted areas. Black scale bars = 200 µm, red scale bars = 50 µm. (**G**) Representative images of the spleen from all groups at 6 w p.i. and from a healthy mouse for comparison. (**H**) Spleen weight from all groups at 6 w p.i. and healthy mice for comparison (n = 3 for all groups). Data are presented as the means +/- SEM. *: $P < 0.05$, **: $P < 0.01$.

Supplementary Fig. 4

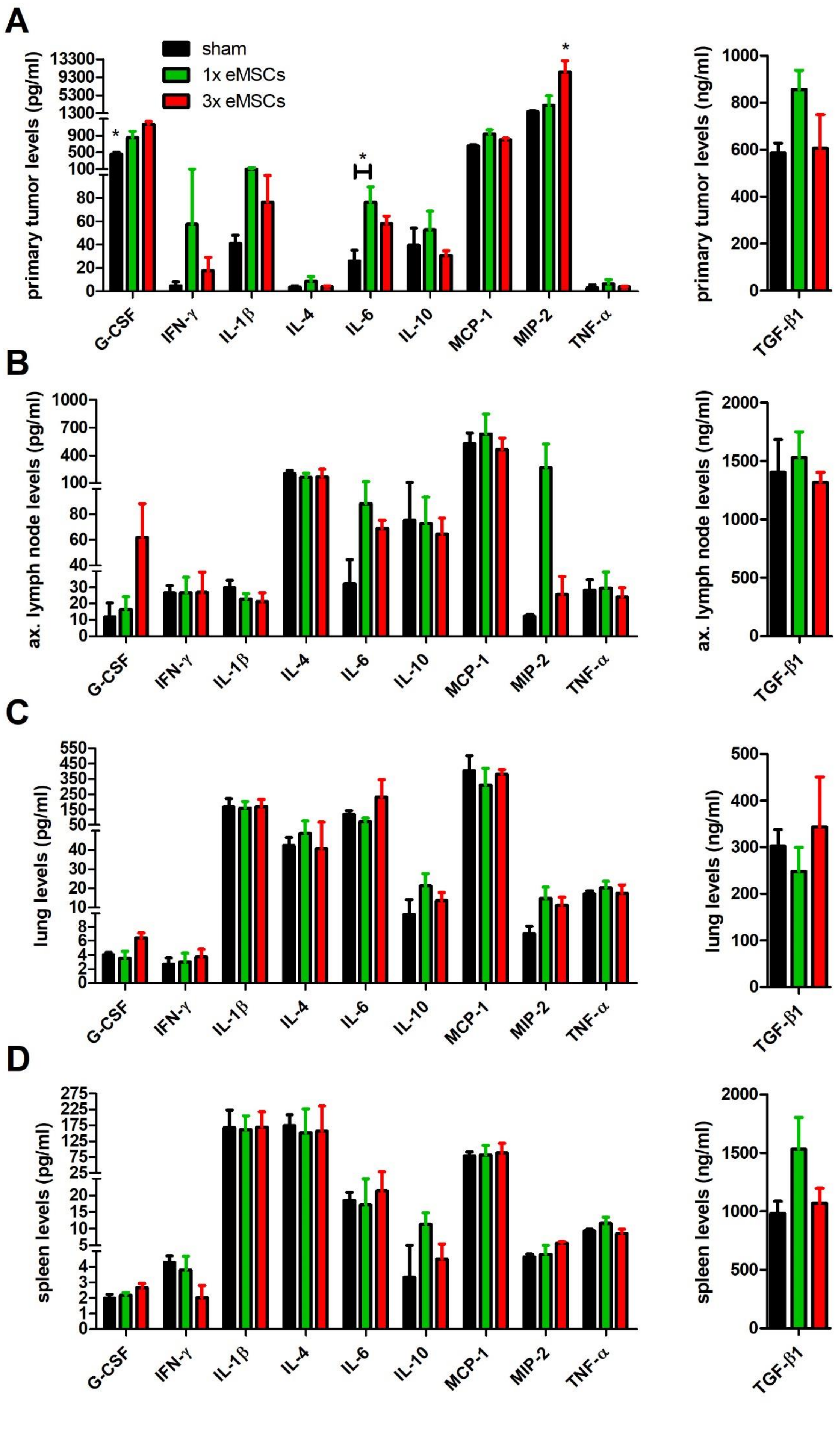

Supplementary Figure 4. Cyto- and chemokine profiles upon eMSC treatment in a 4T1-based intraductal model

(**A-D**) Levels for G-CSF, IFN-γ, IL-1β, IL-4, IL-6, IL-10, MCP-1, MIP-2, TNF-α and TGF-β1 in primary tumors (**A**), axillary lymph nodes (**B**), lungs (**C**) and spleen (**D**) from sham-, 1x eMSC- and 3x eMSC-treated mice at 6 w p.i. (n = 3 for all groups in all tissues, except for the 1x eMSCs group n = 4 in primary tumors). Data are presented as the means +/- SEM. *: $P < 0.05$.

Supplementary Fig. 5

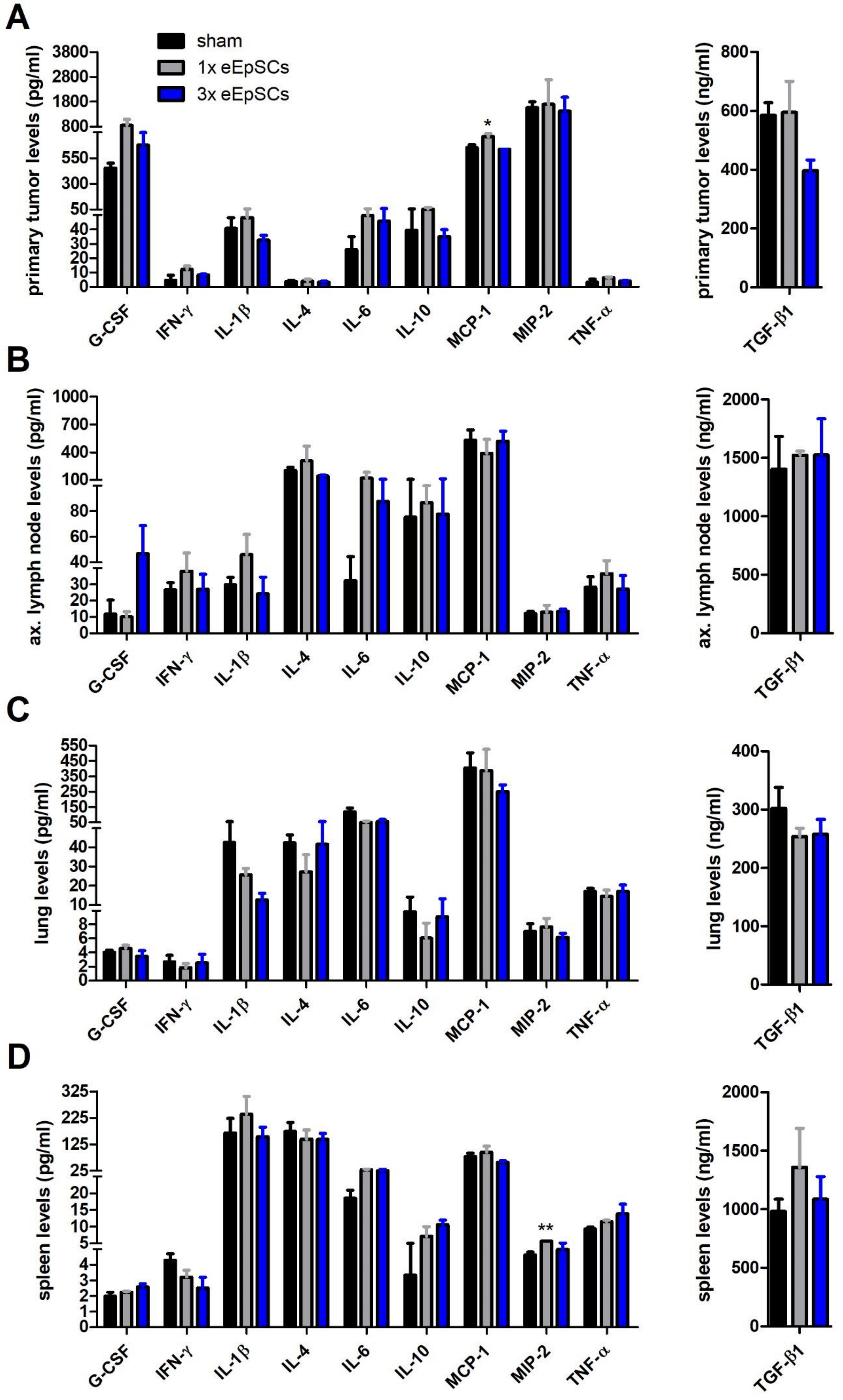

Supplementary Figure 5. Cyto- and chemokine profiles upon eEpSC treatment in a 4T1-based intraductal model

(**A-D**) Levels for G-CSF, IFN-γ, IL-1β, IL-4, IL-6, IL-10, MCP-1, MIP-2, TNF-α and TGF-β1 in primary tumors (**A**), axillary lymph nodes (**B**), lungs (**C**) and spleen (**D**) from sham-, 1x eEpSC- and 3x eEpSC-treated mice at 6 w p.i. (n = 3 for all groups in all tissues). Data are presented as the means +/- SEM. *: $P < 0.05$, **: $P < 0.01$.

Supplementary Fig. 6

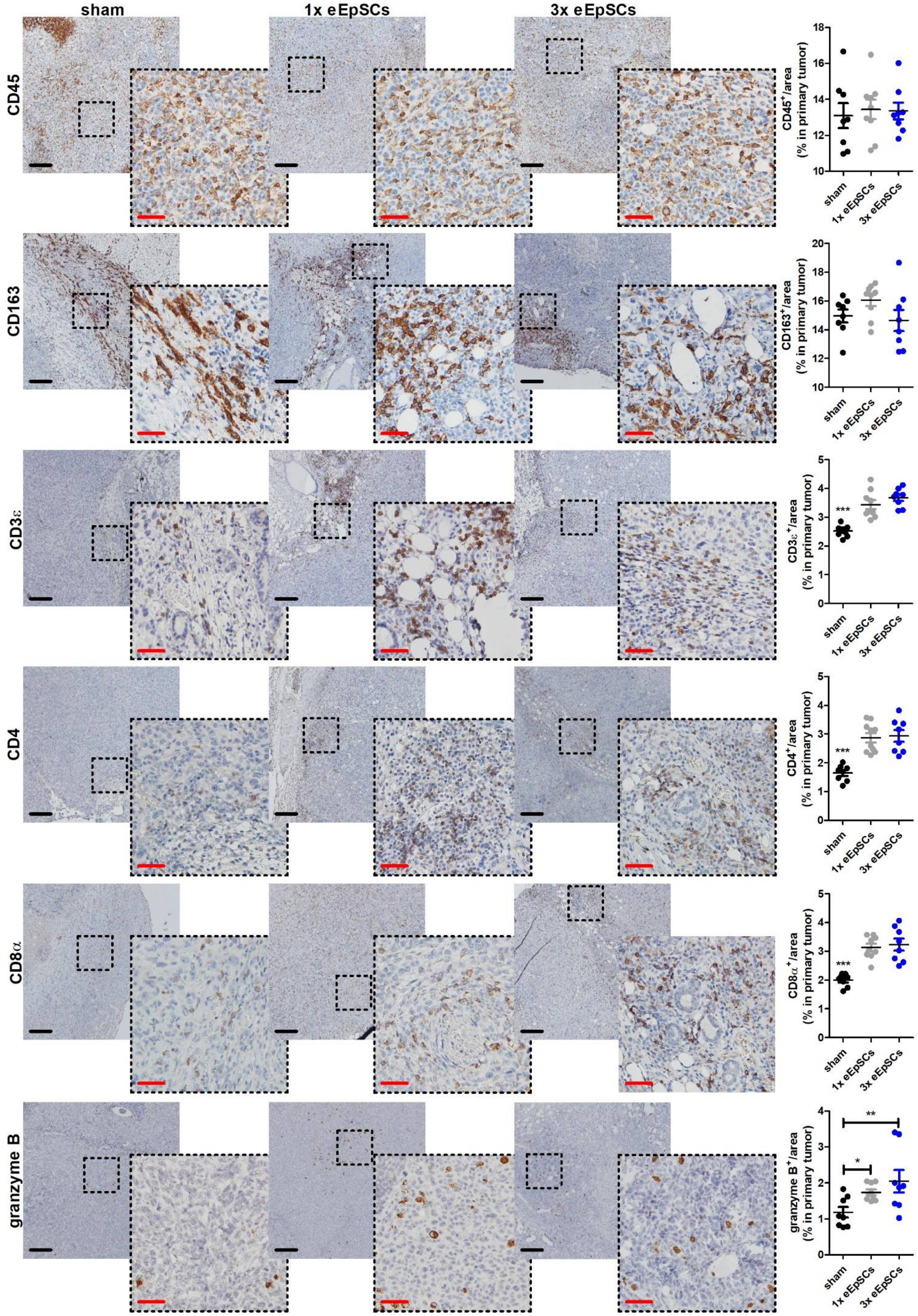

Supplementary Figure 6. Increased T-cell infiltration and activation in primary tumors upon eEpSC treatment in a 4T1-based intraductal model

Immunohistochemistry for the leukocyte marker CD45, the M2 TAM marker CD163, the T-cell marker CD3ε, the specific T-cell subtype markers CD4 and CD8α, and the lymphocytic activation marker granzyme B on primary tumor sections from sham-, 1x eEpSC- and 3x eEpSC-treated mice at 6 w p.i. (n = 8 for all groups; 2 tissue slides with 4 images per slide). Dashed inserts highlight stained tissue at a larger magnification. Black scale bars = 200 µm, red scale bars = 50 µm. Data are presented as the means +/- SEM. *: $P < 0.05$, **: $P < 0.01$, ***: $P < 0.001$.

Supplementary Fig. 7

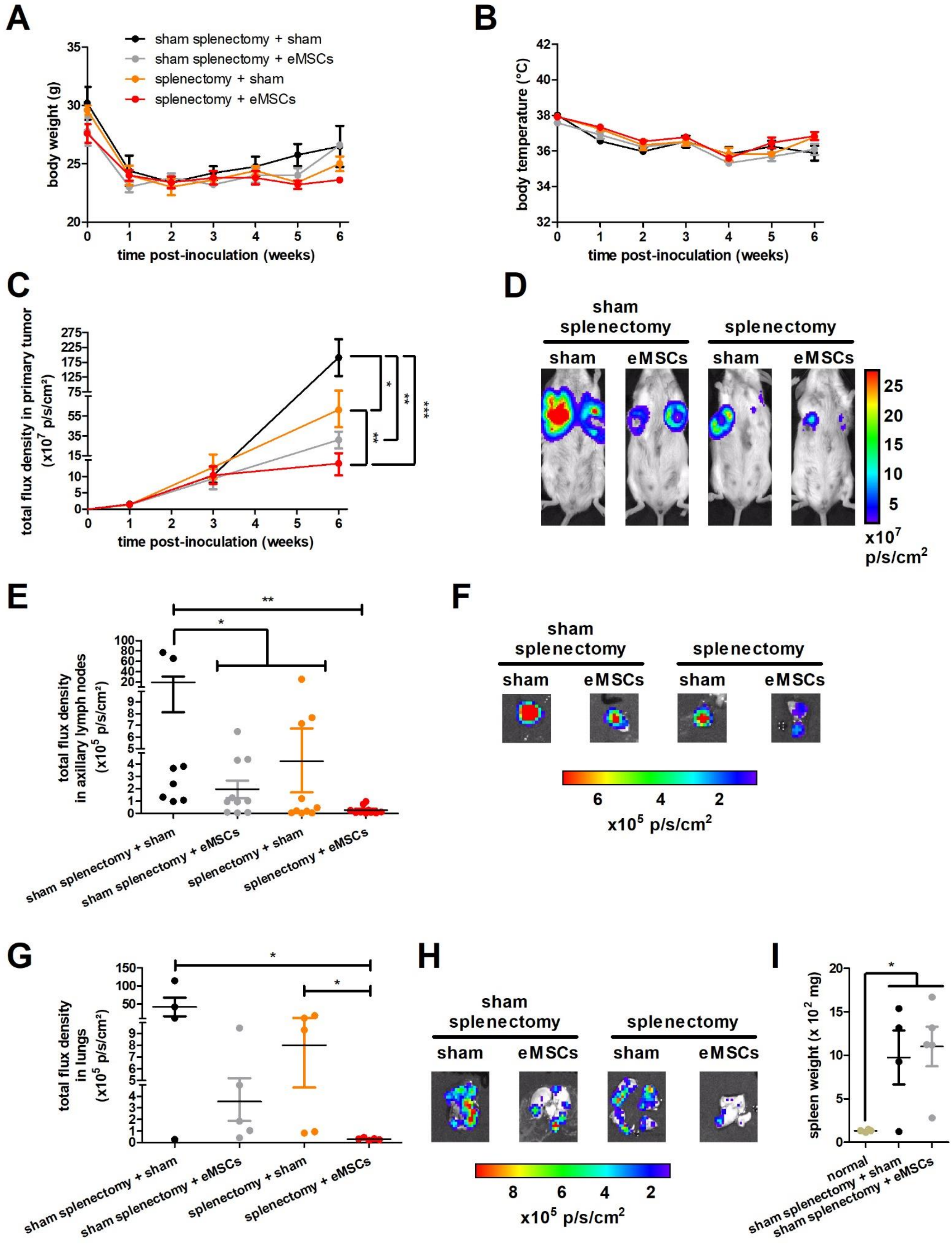

Supplementary Figure 7. Unimpacted eMSC-mediated metastatic reduction in a 4T1-based intraductal model by splenectomy

(**A, B**) Body weight (**A**) and temperature (**B**) across the 6 w study period of (sham) splenectomized mice treated with sham or eMSCs (n = 5 for all groups at all time points, except for the sham splenectomy + sham group at 4, 5 and 6 w p.i. n = 4). (**C**) *In vivo* imaging of bioluminescence signals in the primary tumor areas (shown as total flux density in p/s/cm²; n = 10 for all groups at all time points, except for the sham splenectomy + sham group at 6 w p.i. n = 8). (**D**) Representative images of the primary tumor bioluminescence at 6 w p.i. (**E**) Quantification of 4T1-derived bioluminescence in axillary lymph nodes from all groups at 6 w p.i. based on total flux density (in p/s/cm²) (n = 8 for the sham splenectomy + sham group; n = 10 for all other groups). (**D**) Representative images of bioluminescence in axillary lymph nodes from all groups at 6 w p.i. (**E**) Quantification of 4T1-derived bioluminescence in lungs from all groups at 6 w p.i. based on total flux density (in p/s/cm²) (n = 4 for the sham splenectomy + sham group; n = 5 for all other groups). (**F**) Representative images of bioluminescence in lungs from all groups at 6 w p.i. (**G**) Spleen weight from all groups at 6 w p.i. and healthy mice for comparison (n = 4 for the normal and sham splenectomy + sham group; n = 5 for all other groups). Data are presented as the means +/- SEM. *: $P < 0.05$, **: $P < 0.01$, ***: $P < 0.001$.

Supplementary Fig. 8

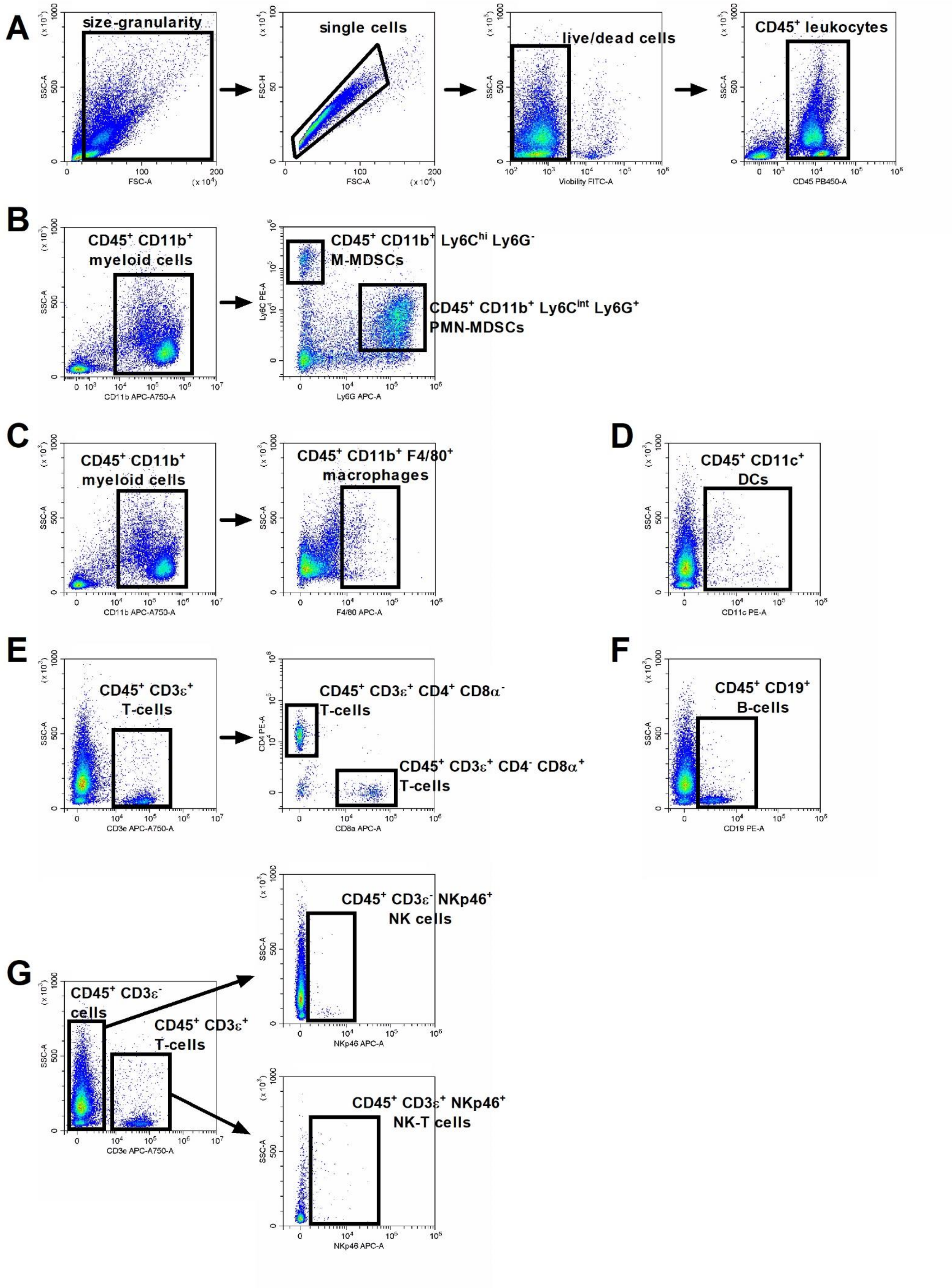

Supplementary Figure 8. Gating strategy for flow cytometric immunophenotyping on a spleen sample from a 4T1-based intraductal model

(**A**) Gating of $CD45^+$ leukocytes following selection of single cells and exclusion of dead cells. The $CD45^+$ gating was also applied in the next panels. (**B**) Gating of $CD45^+$ $CD11b^+$ myeloid cells, with subsequent gating of $CD45^+$ $CD11b^+$ $Ly6C^{int}$ $Ly6G^+$ PMN-MDSC and $CD45^+$ $CD11b^+$ $Ly6C^{hi}$ $Ly6G^-$ M-MDSC subsets. (**C**) Gating of $CD45^+$ $CD11b^+$ myeloid cells, with subsequent gating of $CD45^+$ $CD11b^+$ $F4/80^+$ macrophages. (**D**) Gating of $CD45^+$ $CD11c^+$ DCs. (**E**) Gating of $CD45^+$ $CD3\varepsilon^+$ T-cells, and subsequent gating of $CD45^+$ $CD3\varepsilon^+$ $CD4^+$ $CD8\alpha^-$ and $CD45^+$ $CD3\varepsilon^+$ $CD4^-$ $CD8\alpha^+$ T-cell subsets. (**F**) Gating of $CD45^+$ $CD19^+$ B-cells. (**G**) Gating of $CD45^+$ $CD3\varepsilon^+$ T-cells, and subsequent gating of $CD45^+$ $CD3\varepsilon^+$ $NKp46^+$ NK-T cells. Gating of $CD45^+$ $CD3\varepsilon^-$ cells was also performed for subsequent gating of $CD45^+$ $CD3\varepsilon^-$ $NKp46^+$ NK cells.

Supplementary Fig. 9

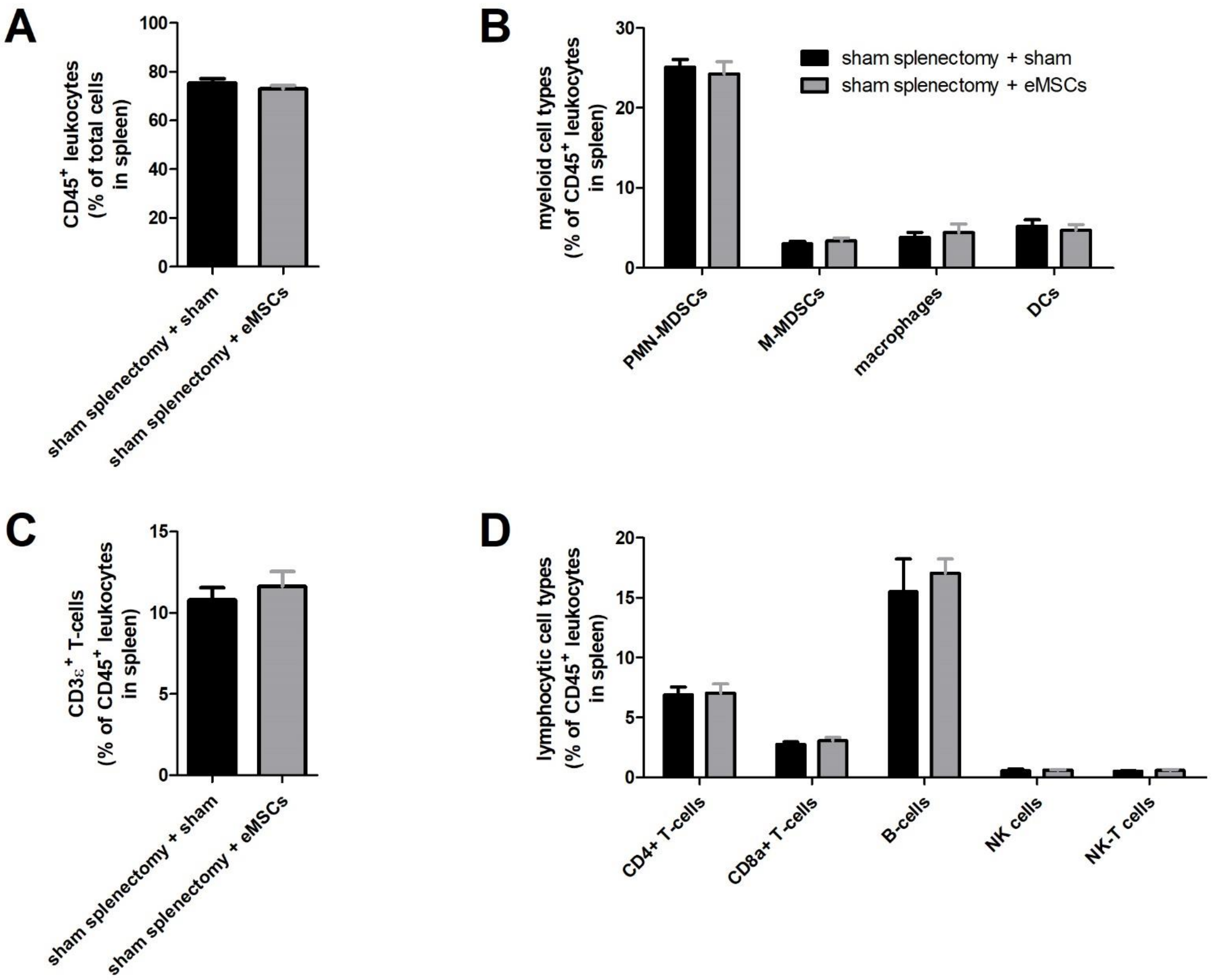

Supplementary Figure 9. Unaffected splenic immune environment by sham or eMSC treatment in a 4T1-based intraductal model

(**A-D**) Flow cytometric immunophenotyping of spleens at 6 w p.i. isolated from sham-splenectomized mice treated with sham or eMSCs (n = 4 for the sham splenectomy + sham group; n = 5 for the splenectomy + sham group). (**A**) Percentage of $CD45^+$ leukocytes within the total cell suspension. (**B**) Percentage of myeloid cell types (including PMN-MDSCs, M-MDSCs, macrophages and DCs) within the $CD45^+$ leukocyte population. (**C**) Percentage of $CD3\varepsilon^+$ T-cells within the $CD45^+$ leukocyte population. (**D**) Percentage of lymphocytic cell types (including $CD4^+$ and $CD8\alpha^+$ T-cells, B-cells, NK and NK-T cells) within the $CD45^+$ leukocyte population. Data are presented as the means +/- SEM.

Supplementary Fig. 10

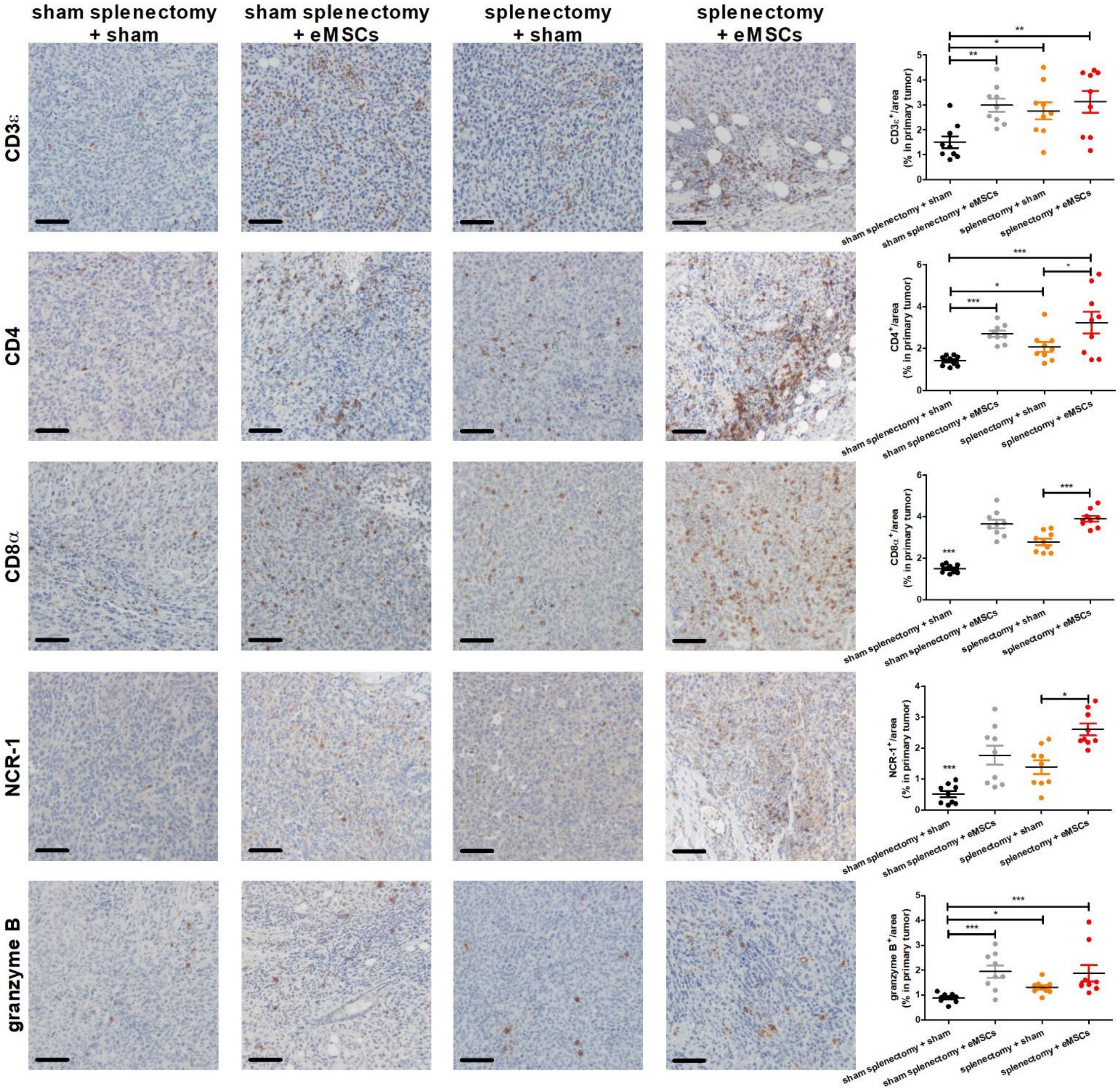

Supplementary Figure 10. Unimpacted eMSC-mediated increase in tumor-infiltrated and activated T-cells by splenectomy in a 4T1-based intraductal model

Immunohistochemistry for the T-cell marker CD3ε, the specific T-cell subtype markers CD4 and CD8α, the NK(-T) cell marker NCR-1, and the lymphocytic activation marker granzyme B on primary tumor sections at 6 w p.i. isolated from (sham) splenectomized mice treated with sham or eMSCs (n = 9 for all groups; 3 tissue slides with 3 images per slide). Scale bars = 50 µm. Data are presented as the means +/- SEM. **: $P < 0.01$, ***: $P < 0.001$.

Supplementary Fig. 11

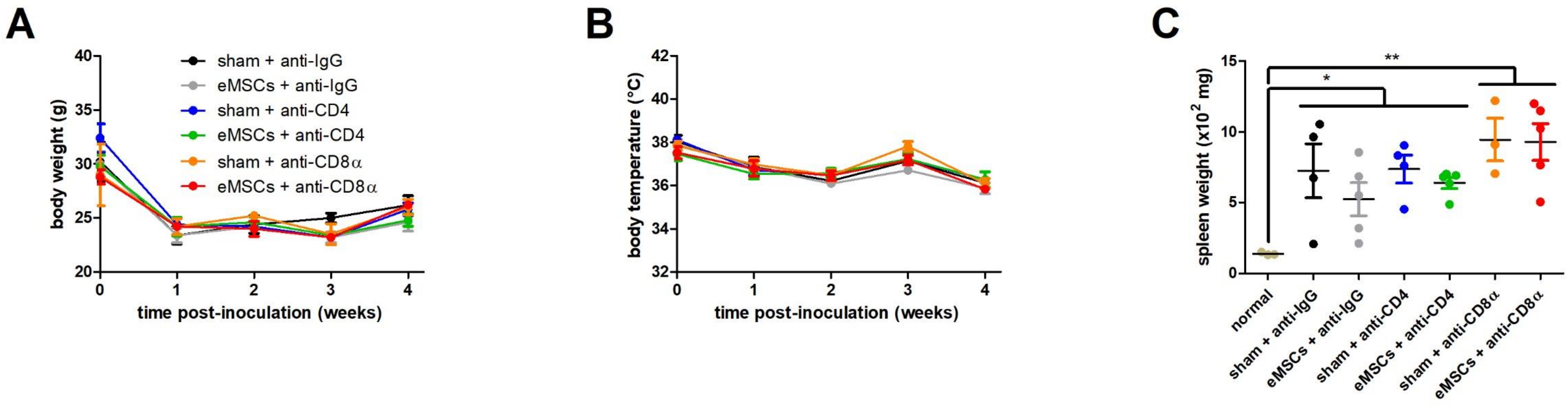

Supplementary Figure 11. Unaffected animal welfare and splenomegaly by eMSC treatment with or without $CD4^+$ and $CD8\alpha^+$ T-cell depletion in a 4T1-based intraductal model

(**A, B**) Body weight (**A**) and temperature (**B**) across the 4 w study period of sham- and eMSC-treated mice following anti-IgG control, -CD4 or -CD8α depletion (n = 5 for all groups at all time points, except for the sham + anti-CD8α group at 3 and 4 w p.i. n = 4). (**C**) Spleen weight from all groups at 4 w p.i. and healthy mice for comparison (n = 5 for the eMSCs + anti-IgG, -CD4 and CD8α group; n = 4 for the sham + anti-IgG and -CD4 group; n = 3 for the normal and sham + anti-CD8α group). Data are presented as the means +/- SEM. *: $P < 0.05$, **: $P < 0.01$.

Supplementary Fig. 12

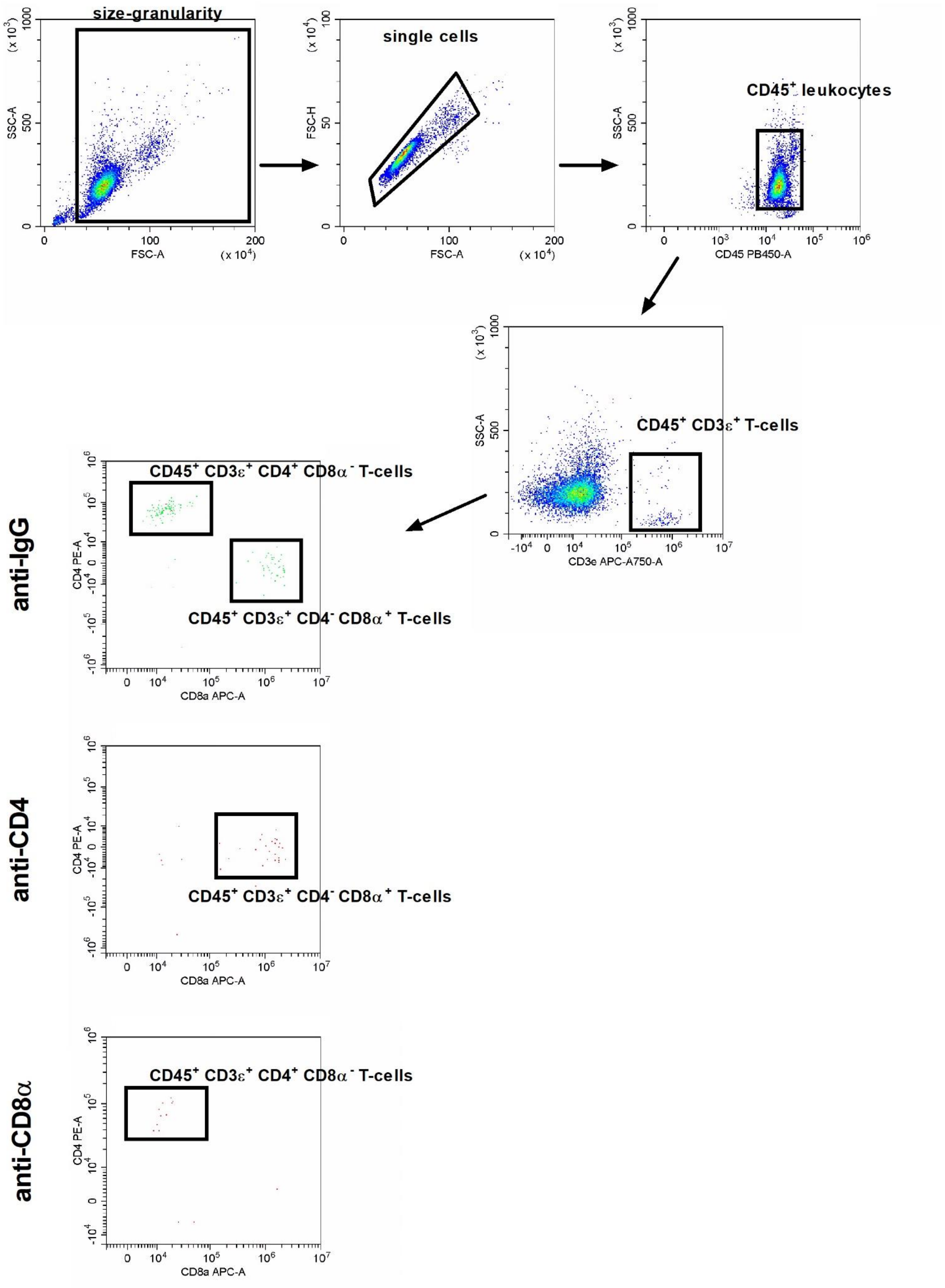

Supplementary Figure 12. Flow cytometric verification of $CD4^+$ and $CD8\alpha^+$ T-cell depletion in blood from a 4T1-based intraductal model

Gating of $CD45^+$ leukocytes following selection of single cells and exclusion of dead cells, allowed subsequent gating of $CD3\varepsilon^+$ T-cells and more specific $CD45^+$ $CD3\varepsilon^+$ $CD4^+$ $CD8\alpha^-$ and $CD45^+$ $CD3\varepsilon^+$ $CD4^-$ $CD8\alpha^+$ T-cell subsets to verify successful T-cell depletion.

Supplementary Fig. 13

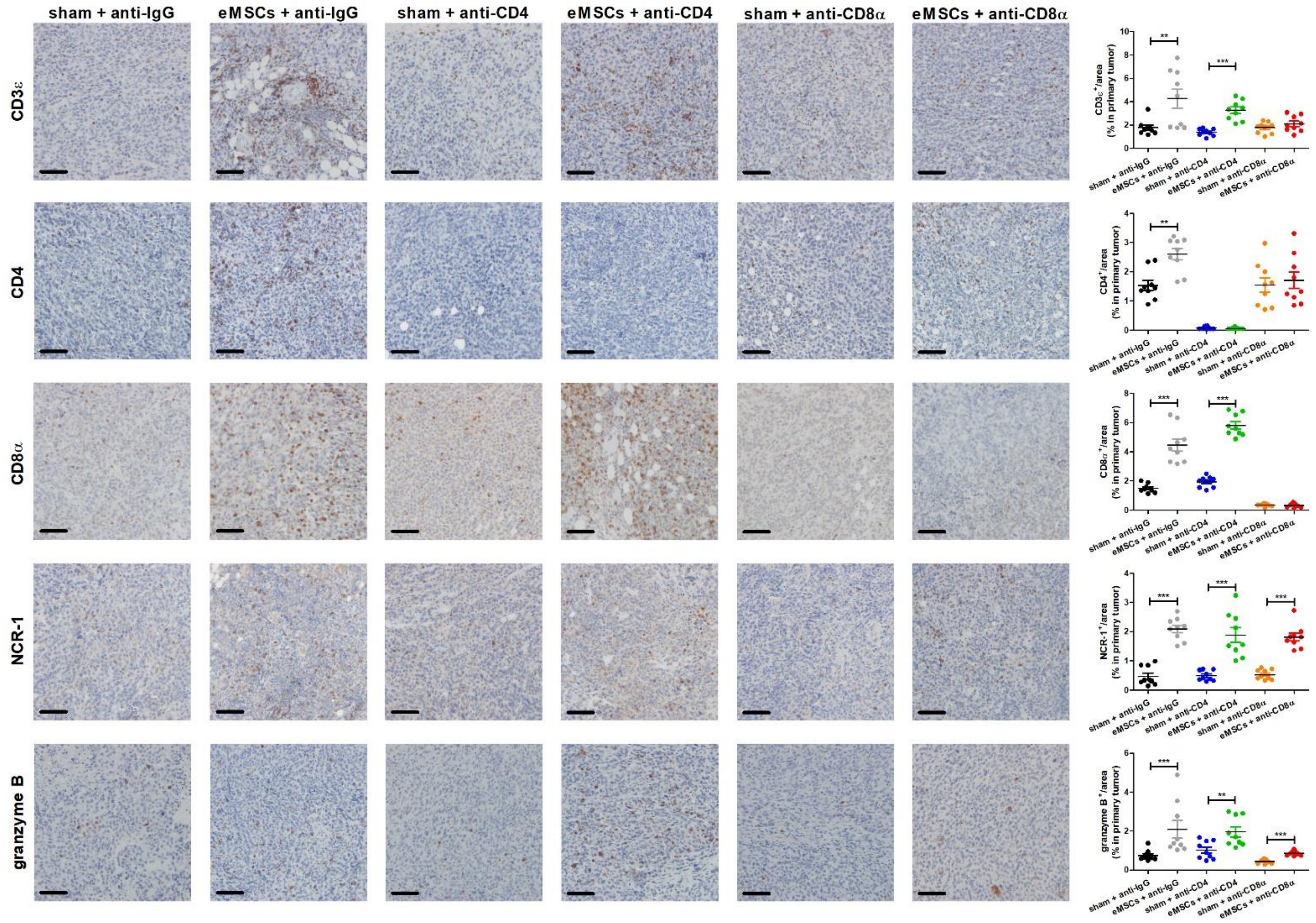

Supplementary Figure 13. Immunohistochemical verification that eMSC-mediated increase in tumor-infiltrated T-cells is affected by CD8α$^+$ T-cell depletion in a 4T1-based intraductal model

Immunohistochemistry for the T-cell marker CD3ε, the specific T-cell subtype markers CD4 and CD8α, the NK(-T) cell marker NCR-1, and the lymphocytic activation marker granzyme B on primary tumor sections at 4 w p.i. isolated from sham- or eMSC-treated mice following anti-IgG control, -CD4 or -CD8α depletion (n = 9 for all groups; 3 tissue slides with 3 images per slide). Scale bars = 50 µm. Data are presented as the means +/- SEM. *: $P < 0.05$, **: $P < 0.01$, ***: $P < 0.001$.
